# Polarized Sonic Hedgehog Protein Localization and a Shift in the Expression of Region-Specific Molecules Is Associated With the Secondary Palate Development in the Veiled Chameleon

**DOI:** 10.3389/fcell.2020.00572

**Published:** 2020-07-28

**Authors:** Marek Hampl, Jana Dumkova, Michaela Kavkova, Hana Dosedelova, Anna Bryjova, Oldrich Zahradnicek, Martin Pyszko, Milos Macholan, Tomas Zikmund, Jozef Kaiser, Marcela Buchtova

**Affiliations:** ^1^Laboratory of Molecular Morphogenesis, Institute of Animal Physiology and Genetics, Czech Academy of Sciences, Brno, Czechia; ^2^Department of Experimental Biology, Faculty of Science, Masaryk University, Brno, Czechia; ^3^Department of Histology and Embryology, Faculty of Medicine, Masaryk University, Brno, Czechia; ^4^Laboratory of Computed Tomography, Central European Institute of Technology, Brno University of Technology, Brno, Czechia; ^5^Institute of Vertebrate Biology, Czech Academy of Sciences, Brno, Czechia; ^6^Department of Developmental Biology, Institute of Experimental Medicine, Czech Academy of Sciences, Prague, Czechia; ^7^Department of Radiation Dosimetry, Nuclear Physics Institute, Czech Academy of Sciences, Prague, Czechia; ^8^Department of Anatomy, Histology, and Embryology, Faculty of Veterinary Medicine, University of Veterinary and Pharmaceutical Sciences, Brno, Czechia; ^9^Laboratory of Mammalian Evolutionary Genetics, Institute of Animal Physiology and Genetics, Czech Academy of Sciences, Brno, Czechia

**Keywords:** secondary palate, SHH, primary cilia, skeletogenesis, chameleon, reptile

## Abstract

Secondary palate development is characterized by the formation of two palatal shelves on the maxillary prominences, which fuse in the midline in mammalian embryos. However, in reptilian species, such as turtles, crocodilians, and lizards, the palatal shelves of the secondary palate develop to a variable extent and morphology. While in most Squamates, the palate is widely open, crocodilians develop a fully closed secondary palate. Here, we analyzed developmental processes that underlie secondary palate formation in chameleons, where large palatal shelves extend horizontally toward the midline. The growth of the palatal shelves continued during post-hatching stages and closure of the secondary palate can be observed in several adult animals. The massive proliferation of a multilayered oral epithelium and mesenchymal cells in the dorsal part of the palatal shelves underlined the initiation of their horizontal outgrowth, and was decreased later in development. The polarized cellular localization of primary cilia and Sonic hedgehog protein was associated with horizontal growth of the palatal shelves. Moreover, the development of large palatal shelves, supported by the pterygoid and palatine bones, was coupled with the shift in *Meox2*, *Msx1*, and *Pax9* gene expression along the rostro-caudal axis. In conclusion, our results revealed distinctive developmental processes that contribute to the expansion and closure of the secondary palate in chameleons and highlighted divergences in palate formation across amniote species.

## Introduction

The morphology of the dorsal area of the oral cavity varies among amniotic groups (Abramyan and Richman, 2015). Reptiles and birds form an incomplete secondary palate with either large openings that connect the oral and nasal cavities or narrow natural clefts, with the exception of crocodilians that develop a fully closed secondary palate (Abramyan et al., 2014). The hard palate is made up of several bones. In mammals, the most rostral part of the hard palate is formed by the premaxillary bone, the largest area is supported by palatal prominences of the maxillary bones, and only the most caudal part of the hard palate is supported by the palatine bones. Compared to mammals, the premaxillary bone in birds is significantly larger and represents the major upper beak forming bone. The largest bones are the palatines, while the maxillary bones are markedly reduced in size. The pterygoid bones in birds are located caudally behind the palatine bones (Richman et al., 2006). In some turtles that develop a secondary palate, the hard palate is formed by the premaxillary, maxillary, and palatine bones, and at the midline by the vomer. In some extinct turtle species, medial prominences of the jugal bones grow into the hard palate (Abramyan and Richman, 2015). In contrast, the arrangement of bones contributing to the hard palate in crocodilians is similar to mammals, except for the most caudal part, which is supported by the ectopterygoid and pterygoid bones.

In chameleons, the hard palate is rostrally formed by the palatine bones and caudally by the pterygoid bones (Romer, 1956). This pattern demonstrates a much larger role for the pterygoid bones in the formation of the palate in reptiles compared to mammals, where the pterygoid is localized caudally from the palatine bone and contributes to the formation of the soft palate (Mohamed, 2018). In comparison to mammals and crocodilians, the maxillary bones of chameleons support the palate only laterally. In the rostral portion of the chameleon palatal midline, the palate is supported by the vomer, which stiffens the nasal septum, and it is located dorsal to the palatal plane. The caudal part is partly formed by the ectopterygoid, which connects the pterygoid, maxillary, and jugal bones (Tolley and Herrel, 2015). Here, we will focus on the initiation of individual skeletal elements that support the hard palate in the chameleon as well as developmental processes that contribute to the formation of their large palatal shelves.

The palatal shelves form as bilateral outgrowth processes from the maxillary prominences during embryonic development in amniotes. They grow in the medial direction toward the midline to either incompletely or fully separate the oral and nasal cavities. During mammalian embryogenesis, the palatal shelves first grow vertically alongside the tongue; later, they are redirected into the horizontal plane to elongate toward each other. This process results in their contact at the midline and fusion with the opposite palatal shelf (Greene and Pratt, 1976; Ferguson, 1988). Crocodilians form a complete secondary palate similar to mammals, but a large proportion of the palatal shelves expands in the horizontal direction from the beginning of the development. Only the most caudal part of their palatal shelves is similar to the mammalian growth pattern, namely the initial vertical growth followed by horizontal course (Ferguson, 1981b). Solely horizontal growth is typical also for lizards and birds. Unlike crododillians, however, the palatal shelves never fuse together in these clades, forming the “physiological palatal cleft” (Ferguson, 1988; Richman et al., 2006; Abramyan et al., 2014).

Most adult chameleons possess open secondary palate with a narrow spacing between the palatal shelves in the midline. However, in some adult animals, the palatal shelves are in contact and form an enclosed secondary palate that resembles the crocodilian palate. Formation of the long palatal processes, which are almost in contact in midline can be caused by a combination of many different morphological aspects, e.g., relative skull dimensions, or tongue shape, as well as their altered size. These can arise as combination of many developmental processes such as enhanced cell proliferation/decreased apoptosis, alteration of cellular polarity etc. In this study, we evaluated in detail some of the developmental processes that contribute to the formation of unique features of the chameleon secondary palate. The macroscopic and microscopic structures of the chameleon palate were analyzed during pre- and post-hatching stages because the growth of the palatal shelves continues in chameleon also during post-hatching developmental period.

## Materials and Methods

### Animals

Embryonic specimens of the veiled chameleon (*Chamaeleo calyptratus*) were obtained from private and commercial breeders. Sample of the Siamese crocodile (*Crocodylus siamensis*) was kindly provided from the collection of prof. Sedmera (Department of Anatomy, Faculty of Medicine, Charles University, Prague, Czech Republic). Specimens of the ocelot gecko (*Paroedura picta*) were obtained from Prof. Lukáš Kratochvíl (Department of Ecology, Faculty of Science, Charles University, Prague, Czech Republic).

Chameleon eggs were purchased from a private breeder (Prague, Czech Republic) and cultivated on moistened vermiculite substrate at 29°C. Each week, starting at week 10 after the oviposition, individuals were collected for immunohistochemistry, RNAScope, and whole-mount *in situ* hybridization (ISH) processing and skeleton staining.

Juvenile and adult reptiles including chameleons were obtained in the frozen state from the University of Veterinary and Pharmaceutical Sciences (Brno, Czech Republic). They were part of the collection at the Department of Anatomy, Histology and Embryology as a gift from Clinics of Small Animals.

The phylogenetic tree was adapted from Hedges (2012) and Zheng and Wiens (2016).

All procedures were performed according to the experimental protocols and rules established by the Laboratory Animal Science Committee of the Institute of Animal Physiology and Genetics (Liběchov, Czech Republic).

### Micro-Computed Tomography (CT) Analysis

Frozen juvenile and adult individuals were measured, dissected to determine gender, and decapitated. We removed the mandible and fixed the head in 4% paraformaldehyde (PFA). The microCT measurements were performed using the GE Phoenix v|tome|x L 240 laboratory system equipped with a 180 kV/15 W nanofocus tube. The measurements were performed at an acceleration voltage of 60 kV and X-ray tube current of 200 μA. The acquisition time was 900 ms for every 2,000 images of a 360° rotation. The microCT data were obtained with a voxel resolution of 4.5 μm. Tomographic reconstruction was realized with the GE phoenix datos|x 2.0 software. Manual segmentation of microCT data and 3D model imaging were implemented in the VG Studio software MAX.

### Alcian Blue and Sirius Red Staining on Slides

Transversal sections were stained with Alcian blue for cartilage and Sirius red for collagen fibers (010254, Diapath, Italy). Histological sections were deparaffinized with xylene and rehydrated through a descending alcohol series. Samples were stained with 1% Alcian blue in 3% acetic acid (10 min), Ehrlich hematoxylin (2 min), 2.5% phosphomolybdic acid (10 min), and 0.1% Sirius Red (1 h).

### Skeleton Staining

Embryos were removed from eggs, decapitated, and the heads fixed in absolute ethanol. The heads were stained with an Alcian blue and Alizarin red solution, and then cleared in KOH/glycerol. Before fixation, the skin from heads was removed for better penetration of staining solution and for better final visualization.

### Scanning Electron Microscopy

Chameleon embryos were fixed in 4% paraformaldehyde, washed in distilled water, and dehydrated through a graded ethanol series (30–100%). Subsequently, the samples were dried using the CPD 030 Critical Point Dryer (BAL-TEC) and shadowed by gold in a metal shadowing apparatus (Balzers SCD040). The samples were observed and photographed with the TESCAN Vega TS 5136 XM scanning electron microscope (Tescan, Czech Republic).

### Transmission Electron Microscopy

The palatal shelves were removed and fixed in 3% glutaraldehyde for 24 h, washed three times in 0.1 M cacodylate buffer, and post-fixed in 1% OsO_4_ solution for 1 h. After washing in cacodylate buffer, the samples were dehydrated in ethanol, followed by acetone, and embedded in Durcupan epoxy resin. Semithin sections were stained with Toluidine blue. Ultra-thin sections (∼60 nm thick) were cut using the Leica EM UC6 ultramicrotome (Leica Microsystems GmbH, Vienna, Austria) and placed on formvar-coated nickel grids. The selected sections, contrasted with lead citrate and uranyl acetate, were observed using the Morgagni^TM^ 268 transmission electron microscope (FEI Company, Eindhoven, Netherlands). Pictures were taken using the Veleta CCD camera (Olympus, Münster, Germany).

### Immunofluorescence on Histological Slides

**FIGURE 1:**
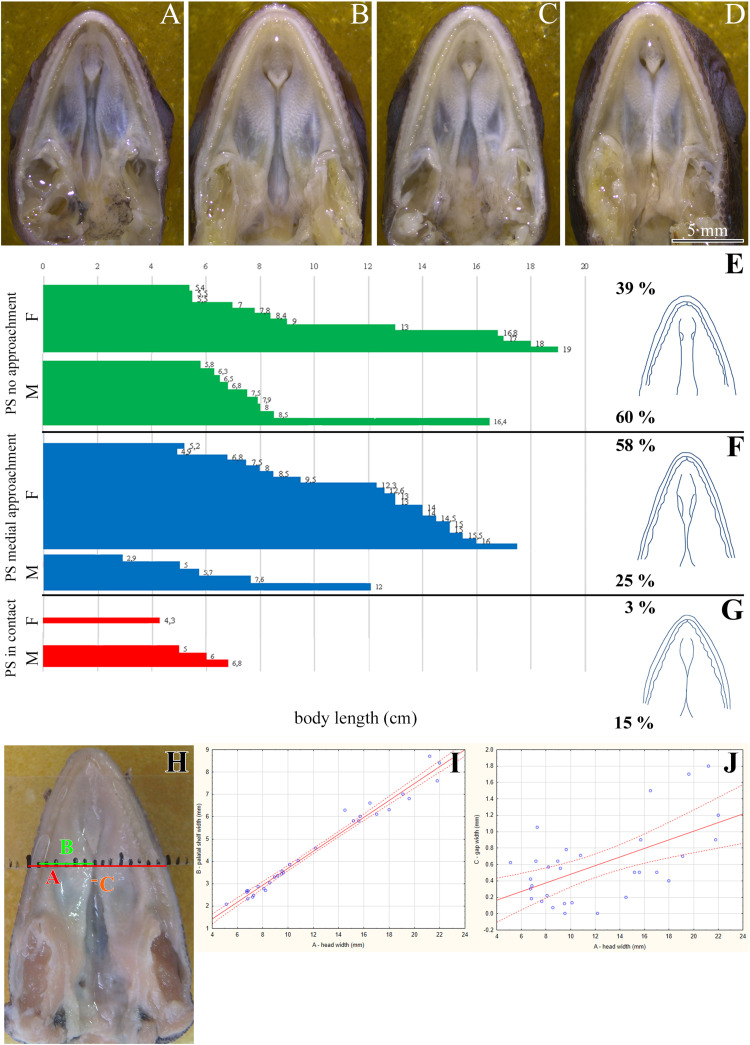
Morphology of the secondary palate in post-hatching stages of the veiled chameleon. Macroscopic view on the palate exhibits different shape and approach state of the palatal shelves in individual chameleons of a similar size **(A–D)**. The graphs compare the distribution of different degrees of a closure of the gap between the palatal shelves in female and male chameleons **(E–G)**. For comparison, 33 female and 20 male adult and juvenile veiled chameleons of different size were used. Head width was measured in place where the distance between the palatal shelves was the smallest (distance **A**, red), and at the same place, width of the palatal shelves was measured from their medial edges to the tooth line (distance **B**, green). In the narrowest place between the palatal shelves the gap was measured (distance **C**, orange). Distance between small black lines in the schema is 1 mm **(H)**. A scatter plot of the palatal shelves width against head width of post-hatching stages **(I)**. Scatter plot of the gap between the palatal shelves against head width of post-hatching stages **(J)**. Thirty one animals were used for measuring the head and palatal shelves size. Ninety five percent confidence regression bands are shown as dashed curves. Scale bar **(A–D)**: 5 mm.

Embryos were removed from eggs, decapitated, and then fixed in 4% PFA overnight. For morphometry, the lower jaw was removed and the palate was photographed. Length and width of the palatal shelves, head widths, and gaps between the palatal shelves, were measured as labeled in Figures 1H, 6E. The scatter plots were created using the Statistica 13.5.0.17 package (TIBCO Software Inc., 2018).

If necessary, the specimens were decalcified before further processing in 12.5% ethylenediaminetetraacetic acid (EDTA) either at room temperature or at 37°C for 1–3 weeks, depending on the calcification level. The specimens were then embedded in paraffin and sectioned (5 μm) in the transversal planes. For immunohistochemistry, the sections were deparaffinized in xylene and rehydrated through descending ethanol series. Antigen retrieval was performed either in 1% citrate buffer or in the DAKO antigen retrieval solution (S1699, DAKO Agilent, United States) at 97.5°C.

For protein localization, we incubated the sections with the primary antibody for 1 h at room temperature or overnight at 4°C. The following antibodies were used: PCNA (1:50, M0879, DAKO Agilent, United States), sonic hedgehog (SHH; 1:100, sc-9024, Santa Cruz, United States), and acetylated α-tubulin (1:200, 32–2700, Invitrogen, Thermo Fisher Scientific, United States). Subsequently, the sections were incubated with the following secondary antibodies (1:200) for 30 min at room temperature: anti-mouse Alexa Fluor 488 (A11001), anti-mouse Alexa Fluor 568 (A11004), anti-rabbit Alexa Fluor 488 (A11008), and anti-rabbit Alexa Fluor 594 (A11037, all Thermo Fisher Scientific, United States).

The sections were mounted with the Prolong Gold antifade mountant with DAPI (P36935, Thermo Fisher Scientific, United States) or Fluoroshield with DAPI (F6057, Sigma, Merck, Germany). If DRAQ5 (62251, Thermo Fisher Scientific, United States) was used for nuclei staining, the sections were mounted with Fluoroshield (F6182, Sigma, Merck, Germany). Pictures were obtained with the Leica DMLB2 fluorescence microscope (Leica, Germany) or Leica SP8 (Leica, Germany) and Zeiss LSM800 (Zeiss, Germany) confocal microscopes. Analysis of polarized localization of SHH ligand was performed using the CirkStat software (author Dr. Lucie Komolíková Burešová).

### Terminal Deoxynucleotidyl Transferase dUTP Nick End Labeling (TUNEL) Assay

Apoptotic cells were detected using the TUNEL assay (ApopTag Peroxidase *In Situ* Apoptosis Detection Kit, Cat. No. S7101, Chemicon, Temecula, United States). Nuclei were counterstained with hematoxylin. The sections were photographed under bright-field illumination with the Leica DMLB2 compound microscope (Leica, Germany).

### Polymerase Chain Reaction (PCR) and Gel Electrophoresis

*Msx1*, *Meox2, Pax9*, and *Hprt1* gene expression was analyzed in tissues isolated from chameleon embryonic bodies. Total RNA was extracted using the RNeasy Plus Mini Kit (74136, Qiagen, Germany) according to the manufacturer‘s instructions. The total RNA concentration and purity was measured using the NanoDrop One (Thermo Fisher Scientific, United States). First-strand complementary DNA (cDNA) was synthesized using the gb Reverse Transcription Kit (3012, Generi Biotech, Czech Republic) according to the manufacturer’s instructions. PCR was performed in 10 μl reactions that contained 10x PCR Buffer + MgCl_2_, PCR Grade Nucleotide Mix, and Fast Start Taq DNA Polymerase (all Roche, Switzerland) mixed with forward and reverse primers for *Msx1*, *Meox2, Pax9*, and *Hprt1* (Generi Biotech, Czech Republic). PCR products were detected using gel electrophoresis in 1% agarose gel at voltage 120 V for 40 min. The primers were designed based on sequences from the veiled chameleon transcriptome (Pinto et al., 2019). Used chameleon sequences of assembled contigs corresponding to individual genes and table with all details are included in Supplementary Material based on published data (Pinto et al., 2019) (Supplementary Material S1).

### Plasmid Purification and *in situ* Hybridization Probe Synthesis

Total RNA was isolated from embryonic chameleon tissues using the RNeasy Plus Mini Kit, according to the manufacturer‘s instructions. The total RNA concentration and purity was measured using the NanoDrop One. First-strand cDNA was synthesized using the gb Reverse Transcription Kit, according to the manufacturer’s instructions. PCR was performed in a 50 μl reaction volume containing 10x PCR Buffer + MgCl_2_, PCR Grade Nucleotide Mix, and Fast Start Taq DNA Polymerase mixed with forward and reverse primers for *Msx1* and *Meox2* (Generi Biotech, Czech Republic). The PCR products were isolated from agarose gels using the QIAquick Gel Extraction Kit (28706, Qiagen, Germany), according to the manufacturer‘s instructions. *Msx1* and *Meox2* PCR products were sequenced (Eurofins, Czech Republic) and used to transform the One Shot MAX efficiency DH5α Competent Cells (44-0097, Thermo Fisher Scientific, United States). The transformed cells were subsequently selected using the TOPO TA Cloning Kit (45-0640, Thermo Fisher Scientific, United States) on Luria-Bertani (LB) agar plates treated with X-gal. Plasmids were then purified using the QIAGEN Plasmid Midi Kit (12145, Qiagen, Germany), according to the manufacturer’s instructions. DNA was sequenced using the endogenous M13F primer site (Eurofins, Czech Republic) and linearized by PCR using both M13 primers. Linearized PCR products were transcribed with the T7 polymerase for the antisense probe (10881767001, Roche, Switzerland) or Sp6 polymerase for the sense probe (10810274001, Roche, Switzerland).

### Whole-Mount *in situ* Hybridization

Whole chameleon embryos or heads were fixed in 4% PFA overnight at 4°C. The tissues were dehydrated and rehydrated in a methanol series. Proteinase K (10 μg/ml) was applied for 45 min at room temperature, and the tissues were then post-fixed in a combination of 4% PFA and 25% glutaraldehyde for 20 min at room temperature. The tissues were incubated with the probe in a hybridization mix at 68°C for *Msx1* and at 60°C for *Meox2* overnight while rotating in a hybridization oven (Compact Line OV4, Biometra, Germany). Subsequently, the tissues were incubated in a hybridization mix, washed with maleic acid buffer containing Tween 20 (MABT), and incubated with anti-digoxigenin (DIG) conjugated to alkaline phosphatase (AP; 11093274910, Roche, Switzerland) overnight at 4°C while shaking. Finally, the signal was developed using the BM Purple (11442074001, Roche, Switzerland). Pictures were captured using the Leica M205 FA stereoscope (Leica, Germany).

### RNAScope

The embryos were fixed in 4% PFA and fixation time differed based on the stage. The tissues were then dehydrated in an ethanol series, embedded in paraffin, and 5 μm transverse sections were obtained. The sections were deparaffinized in xylene and dehydrated in 100% ethanol. To detect gene expression, we used the RNAScope^®^ Multiplex Fluorescent v2 Assay kit (323 110, ACD Bio, United States) for formalin-fixed paraffin embedded tissues according to the manufacturer‘s instructions. All reactions, which require 40°C incubation temperature, were performed in the HybEZ^TM^ II Oven (ACD Bio, United States). The probes were designed based on sequences from the chameleon transcriptome (Supplementary Material); *Msx1* (805271, ACD Bio, United States) and *Meox2* (805281, ACD Bio, United States) probes were used. The hybridized probes were visualized using the TSA-Plus Cyanine 3 system (NEL744001KT, Perkin-Elmer, United States), according to the manufacturer’s protocol. DAPI (323 108, ACD Bio, United States) was used to stain nuclei. Pictures were obtained with the Leica SP8 confocal microscope (Leica, Germany).

### Real-Time PCR

*Msx1*, *Meox2*, and *Pax9* gene expression was analyzed in tissues isolated from the rostral and caudal parts of the palatal shelves and upper jaws. Rostral parts of the palatal shelves included the palatine bones and mesenchyme, caudal palatal shelves contained pterygoid and ectopterygoid bones and adjacent mesenchyme. Rostral parts of the upper jaw contained the maxillary bones and mesenchyme, caudal parts of the upper jaw contained caudal parts of the maxillary bones and rostral part of the jugal bones and adjacent mesenchyme. One sample was pooled from two or three embryos; six biological replicates were analyzed. RNA isolation, and first-strand cDNA synthesis were performed as described above. Real-time PCR reactions were performed using the SYBR Green (3005, Generi Biotech, Czech Republic) in 20-μl reactions on the LightCycler 480 (Roche, Switzerland). The comparative C_*T*_ method was used for analysis. The thermal conditions were as follows: preincubation at 95°C for 10 min, 45 cycles (denaturation at 95°C for 10 s, annealing at 57°C for 10 s, and extension at 72°C for 10 s), and melting at 95°C for 5 s and at 65°C for 1 min. *Hprt1* was used as a housekeeping gene.

## Results

### The Palatal Shelves in Adult Chameleons Are Well Developed and Resemble the Crocodilian Secondary Palate Before Its Closure

Most adult chameleons possess open secondary palate with the large palatal shelves and small spacing between them in the midline. Therefore, the connection between the oral and nasal cavity remains open, similarly to other non-crocodilian reptiles. However, the macroscopic analysis of 53 juvenile and adult individuals of the veiled chameleon (Figures 1A–D), revealed that the lateral palatal shelves are well developed and significantly protruded horizontally toward the midline. The medial edge shape of the palatal shelves can vary from round to flat in both females and males (Figures 1E–G). We also observed that the growth of the palatal shelves continued even during post-hatching stages. This phenomenon can lead to contact or even enclosure of the contralateral shelves at the midline in some adult animals. Enclosure of the secondary palate was observed in several animals of both sexes and regardless of age, with more fused shelves observed in males (15% in males and 3% in females; Figure 1G). However, the majority of male adult animals possessed palatal shelves that did not approach each other (60% in males and 39% in females; Figure 1E). The secondary palate that featured a medial meeting of the palatal shelves was the major pattern for adult female animals (25% in males and 58% in females; Figure 1F).

Because of the diverse extent to which the palatal shelves have developed in individual adults, we tested the correlation (Figures 1H–J) between width of the palatal shelves and width of the head (Figure 1I), and the correlation between width of the gap between the palatal shelves and width of the head (Figure 1J). Indeed, the measurements were found to be highly correlated (*R*^2^ = 97.6%; *p* ≪ 0.0001; and *R*^2^ = 33.7%; *p* < 0.001, respectively).

### Palatal Shelves Are Supported by Skeletal Elements Contributing to Hard Palate Formation

**FIGURE 2:**
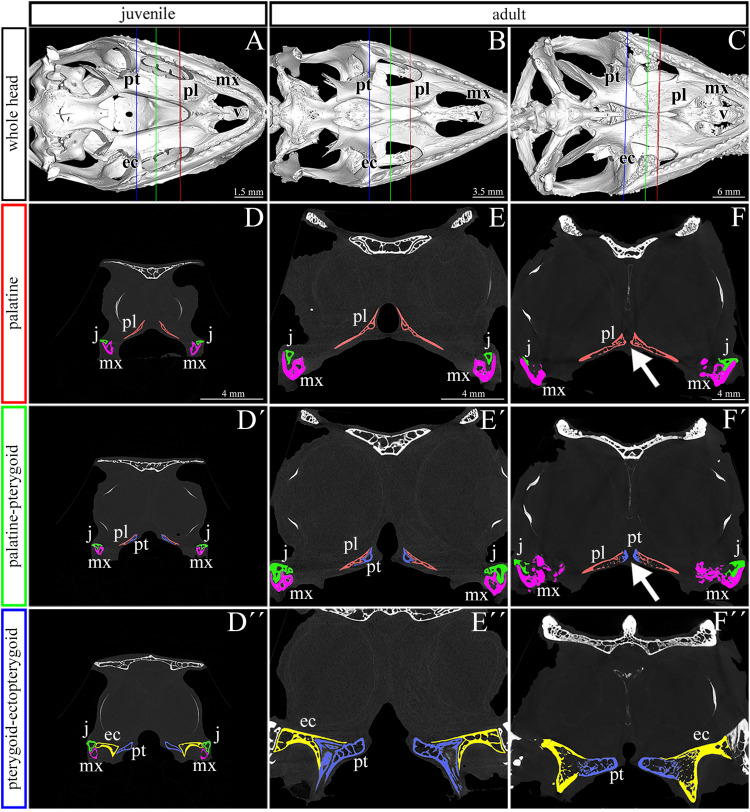
Skeletal analysis of the secondary palate at post-hatching stages of chameleon by microCT. Whole mount cranial skeleton from palatal view displays post-hatching development of the palate-forming bones in chameleon **(A–C)**. Transversal sections of microCT scans reveal developmental and morphological changes of the palate-forming bones in three different planes during post-hatching development. Cross sections through the palatine bones (red lines in whole mount skulls) **(D–F)**. Cross sections of the junction between the palatine and pterygoid bones (green lines on whole mount skulls) **(D′–F′)**. Cross sections of the pterygoid, ectopterygoid, and jugal bones (blue lines on whole mount skulls) **(D″–F″)**. White arrows in **(F,F′)** show contact of soft palatal tissues. ec, ectopterygoid; j, jugal; mx, maxillary bone; pl, palatine bone; pt, pterygoid bone. Scale bar: cross sections 4 mm.

We next used a microCT analysis to reveal, whether enclosure of the palatal shelves was only superficial, i.e., formed by soft tissues, or if bones forming the palatal shelves were in the direct contact (i.e., whether a suture between the bones was formed). We compared different post-hatching stages, from juvenile with largely opened palate (Figure 2A), through adults with large lateral palatal shelves with the initial contact of soft tissues (Figure 2B), to older animals with enclosed secondary palates (Figure 2C). This analysis revealed that the major palate-forming bones (palatine and pterygoid) expanded medially toward the midline during the post-hatching period (Figure 2). However, even in the apparently compact and sealed secondary palate of the oldest animals, the bones were neither fused nor in contact. Therefore, only the soft tissue contributed to palatal shelf fusion (Figures 2F–F″, arrows), a finding that is in contrast to suture formation in crocodiles and mammalian species.

### Thickness of Palate-Forming Bones Changes During Post-hatching Development

**FIGURE 3:**
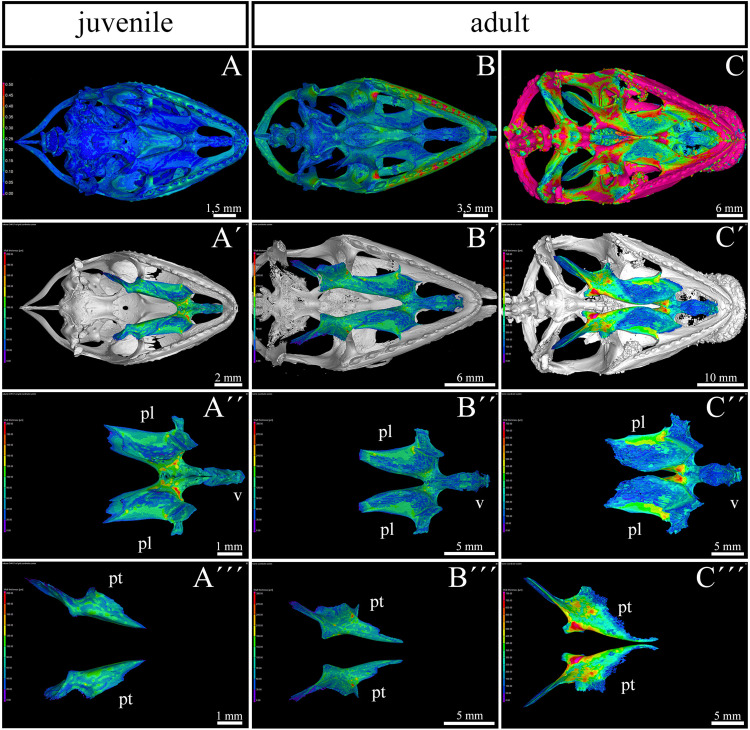
Bone thickness skeletal analysis of individual bones at the post-hatching stages of chameleon by microCT. Bone thickness analysis of juvenile and two adult animals with palatal view on the skull. Same scales were used to reveal differences in ossification progress of individual skeletal elements with the age of animal **(A–C)**. Whole mount cranial skeleton from palatal view displays segmentation of the main palate-forming bones **(A′–C′)**. Detailed segmentation of the palatine bones and vomer in juvenile **(A″)**, younger adult **(B″)** and older adult **(C″)** chameleons. Individually segmented pterygoid bones are shown on **(A″′)** for juvenile, **(B″′)** for younger adult, and **(C″′)** for older adult. Notice that scales in the wall thickness analysis of individual bones **(A′–C″′)** were set individually for each sample in order to describe the minute differences in bone thickness inside of individual skeletal elements. The main reason for setting the scales individually was that the images with unified scale lost some important detail in juvenile or in older adult. Bone thickness is displayed by a different colors from blue (the thinnest, 0 μm) to red (the thickest, up to 200 μm) demonstrated in colors legend. pl, palatine bone; pt, pterygoid bone; v, vomer. Scale bars are displayed individually for each picture.

To evaluate areas with the greatest thickness and expanded growth of bone matrix inside individual skeletal elements contributing to the secondary palate, we performed a wall thickness analysis on micro-CT 3D data (Figure 3). We expected that the bone mass will be remodeled depending on the load on individual skeletal elements during post-hatching development.

The analysis revealed the highest values in the ectopterygoid bone for all analyzed individuals (Figures 3A–C). In the maxillary bone, the thickest area was in the maxillary caudal zone in the juvenile (Figure 3A). The central areas of the maxillae, especially their interdental spaces, exhibited an enhanced wall thickness in older individuals (Figure 3B). In the oldest specimen, the pterygoid bone and the lateral portion of the palatine bone displayed higher values compared to other bones that contribute to the palatal region (Figure 3C).

### The Main Palate-Forming Bones Are the First to Ossify in the Craniofacial Skeleton

**FIGURE 4:**
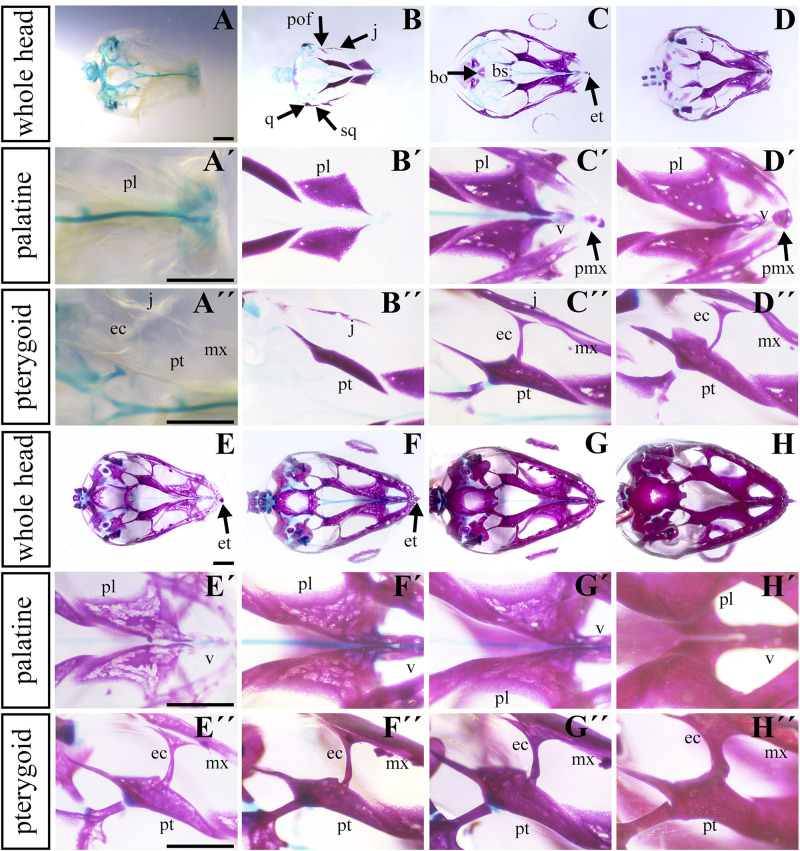
Ossification of individual bones contributing to the secondary palate during pre-hatching stages. Lower power images **(A–D)** of palatal view of all bones and cartilages that contribute to formation of palate at earlier pre-hatching stages of chameleon embryos. Higher power images display either the palatine bones **(A′–D′)** or pterygoid bones **(A″–D″)** ossification during pre-hatching development of the veiled chameleon. The same arrangement of pictures for later stages of pre-hatching stages of chameleon embryos **(E–H″)**. Bones and cartilage were stained using Alcian blue and Alizarin red staining. bo, basioccipital bone; bs, basisphenoid bone; ec, ectopterygoid bone; et, egg tooth; j, jugal bone; mx, maxillary bone; pl, palatine bone; pmx, premaxillary bone; pof, post-orbitofrontal bone; pt, pterygoid bone; q, quadrate bone; sq, squamosal bone; v, vomer. Scale bar: 1 mm.

To uncover the ossification sequence, we performed whole mount skeletal staining during pre-hatching development. Prior to bone mineralization, craniofacial cartilages were present (Figures 4A–A″). The first mineralized skeletal elements in the facial region were the palatine and pterygoid bones (Figures 4B,B″ and Supplementary Figure S1A–A″), slightly followed by the jugal bone in the caudal region (Figure 4B″). Maxillary bones were mineralized later (Figures 4C,C′ and Supplementary Figures S1B,B′) and ossification subsequently proceeded rostrally to connect with the premaxilla and caudally to the joint with the jugal bone (Figure 4C). The progress of bone mineralization resulted in medial contact between the maxillary and palatine bones and rostral contact with the ossifying premaxilla. Concurrent with premaxilla ossification, we observed the development of hard tissues in the egg tooth (Figures 4C′,D′) and the onset of vomer mineralization (Figures 4C′,D′). Caudally, the ossification of the ectopterygoids and the lateral contact of pterygoids and jugal bones was initiated at almost the same time as the ossification of rostral portion of the maxillary bone (Figures 4C–C″).

Simultaneously with ossification centers of the jugal, post-orbitofrontal, squamosal, and quadrate bones started to ossify (Figure 4B), bones of the most caudal craniofacial area, basisphenoid and basioccipital, formed via endochondral ossification. Their mineralization was established just after the ossification of the major palate-forming bones (Figures 4C,D). Their ossification progressed toward each other to form an enclosed basicranium later in development (Figures 4E–H). During this process, the surrounding craniofacial bones continuously mineralized, and the original cartilaginous articulation between them ossified (Figure 4H).

### The Rostral Area of the Palatal Shelves Is Formed by Cartilage

**FIGURE 5:**
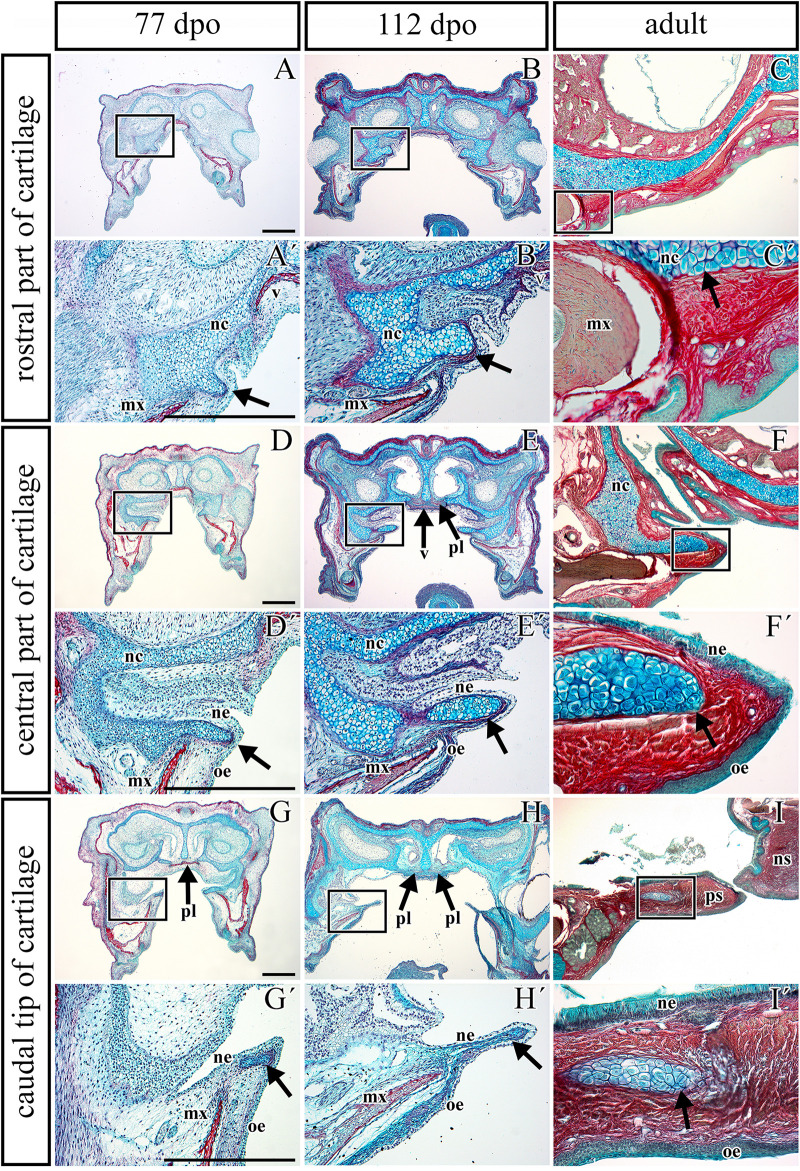
Transversal microscopic sections through the rostral part of head in pre- and post-hatching chameleons with skeletal staining. Alcian blue, Green trichrome, and Sirius red staining on transversal sections display contribution of the palatal cartilage process to the secondary palate formation during pre-hatching development (77 and 112 dpo) and in adult chameleon. Palatal cartilage, as a part of the nasal capsule cartilage, penetrates the palatal shelves at their very rostral parts **(A,A′-C,C′)**, then supports the palatal shelves along the mediolateral axis **(D,D′–F,F′)**. Almost at the middle area of the palatine bones along the rostro-caudal axis, only rudiments of this cartilage are visible in the tip of the palatal shelves **(G,G–I,I′)**. Higher power pictures **(A′–I′)** show details from black rectangles in a lower power pictures **(A–I)**. mx, maxillary bone; nc, nasal cartilage; ne, nasal epithelium; ns, nasal septum; oe, oral epithelium; pl, palatine bone; ps, palatal shelf; v, vomer. Scale bar: 200 μm.

The palatal shelves in birds and mammals are typically only supported by membranous bones (Richman et al., 2006). However, in chameleons, we observed cartilage in the rostral palatal area throughout both pre-hatching and post-hatching (adult) developmental stages. At the pre-hatching developmental stages (77 dpo and 112 dpo), rudimentary cartilage was present in the rostral area of the palatal shelves closely adjacent to the dorsal portion of the palatal shelves. This cartilage expanded into the most medial tips of the palatal shelves (Figures 5A,B) and formed the palatal part of the nasal cartilage. The cartilage protruded between the maxillary bones, located ventrolaterally, and the vomer, located dorsomedially (Figures 5A,A′,B,B′). At the later stage (112 dpo) at the interface of the vomer and palatine bones (Figure 5E, arrows), the cartilage divided into two parts and was covered by the epithelium (Figure 5E′). In this zone, the most rostral area of the palatal shelves protrusion was visible (Figures 5D′,E′), and the ventral part of the separated cartilage protruded medially to form the palatal shelves (Figures 5D′,E′, arrows).

**FIGURE 6:**
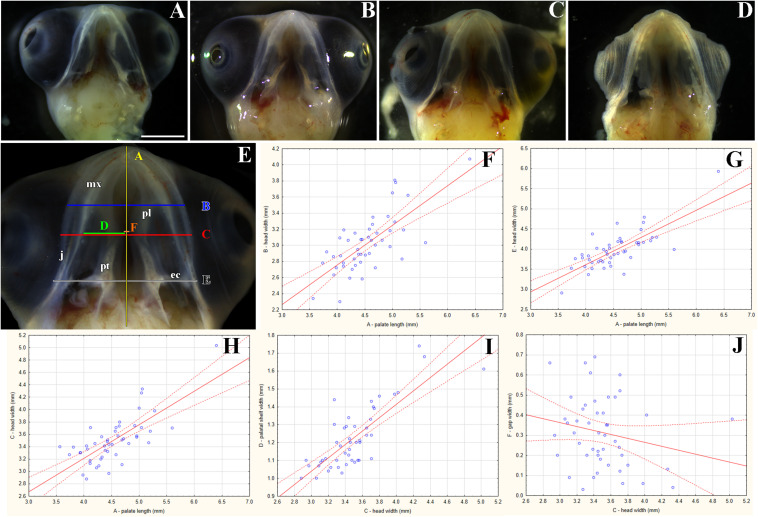
Morphological variation of the palatal shelves in chameleon embryos. Variability of the palatal region in embryonic chameleons within 15th week of the pre-hatching development **(A–D)**. Embryonic heads are aligned from the smallest **(A)** to the largest **(D)**. Head lengths were measured from the tip of the rostrum to the most caudal part of the pterygoid bones (distance **A**); the measurements in panel **(E)** were used for correlation analyses between individual parameters **(F–J)**. Head width and palatal shelves width are shown in millimeters. 48 embryos were analyzed. Ninety five percent confidence regression bands are shown as dashed curves. ec, ectopterygoid; j, jugal bone; mx, maxillary bone; pl, palatine bone; pt, pterygoid bone. Scale bar **(A–D)**: 2 mm.

In the caudal direction, the cartilage gradually retreated from the palatal shelves and was only preserved in the most distal tip (Figures 5G,H). At the earlier embryonic stage (77 dpo), the most caudal part of the cartilage (Figure 5G′, arrow) was located in the area of the palatine bones dilatation (Figure 5G, arrow). At 112 dpo, the cartilage in the tips of the palatal shelves terminated (Figure 5H′, arrow) at the anterior portion of the palatine bones (initial narrow part located medially posterior from vomer; Figure 5H). The same cartilage arrangement was visible in the adult chameleon (Figures 5C–I′).

### Morphogenesis of the Palatal Shelves During Development

During early pre-hatching development (77 dpo), the palatal shelves were initiated as medial bulge-like protrusions of the maxillary prominences (Figures 5A,A′, see also in Figure 8). The dorsal parts of these protrusions were then transformed into long and thin shelves that protruded dorsomedially (Figures 5B,B′). In pre-hatching stages, we did not observe any chameleon with the palatal shelves in direct contact in the midline or with the nasal septum. However, we observed this contact later in development, when the shelves significantly elongated in the dorsomedial direction to meet each other at the midline (Figure 5I).

Like in adults, the size and shape of the palatal structures were highly variable (Figures 6A–D). Nevertheless, individual measurements were strongly correlated (*R*^2^ ranging from 51.8 to 64.4%; *p* < 0.0001; Figures 6F–I). However, a comparison between the gap width and the head width indicated slight, though insignificant, negative correlation (*R*^2^ = 4.5%; *p* = 0.1466; Figure 6J). As expected, the same negative (and marginally significant) correlation was found between the gap width and the palatal shelf width (*R*^2^ = 12.9%; *p* = 0.0122; not shown). This suggests, again rather expectedly, that as the head and upper jaw increase, the gap between the two palatal shelves is progressively closing.

Scanning electron microscopy revealed a thickened structure at the edge of the palatal shelves that resembled the ectodermal ridge of an early limb. The ridge expanded up to half of the palatal shelves and was observed at all three analyzed pre-hatching developmental stages, which corresponded to the age between 17 and 20 weeks (Supplementary Figure S2). The most rostral area that surrounded the primary choanae was rough, with more distinct protuberances formed later in development (Supplementary Figure S2). Numerous microvilli and microplicae developed on the palatal surface (Supplementary Figure S2). In the most caudal area, we observed clusters of motile cilia (Supplementary Figure S2).

### Epithelium of the Palatal Shelves Undergoes Region-Specific Differentiation During Pre-hatching Development

Transmission electron microscopy was used to further analyze the ultrastructure of epithelial cells in detail during development and to determine differences in individual area along the palatal shelves. Moreover, we focused on the ridge located in the tip of the palatal shelves, which we discovered by SEM (Supplementary Figure S2). Two distinct developmental stages (126 dpo and 161 dpo respectively) were selected.

**FIGURE 7:**
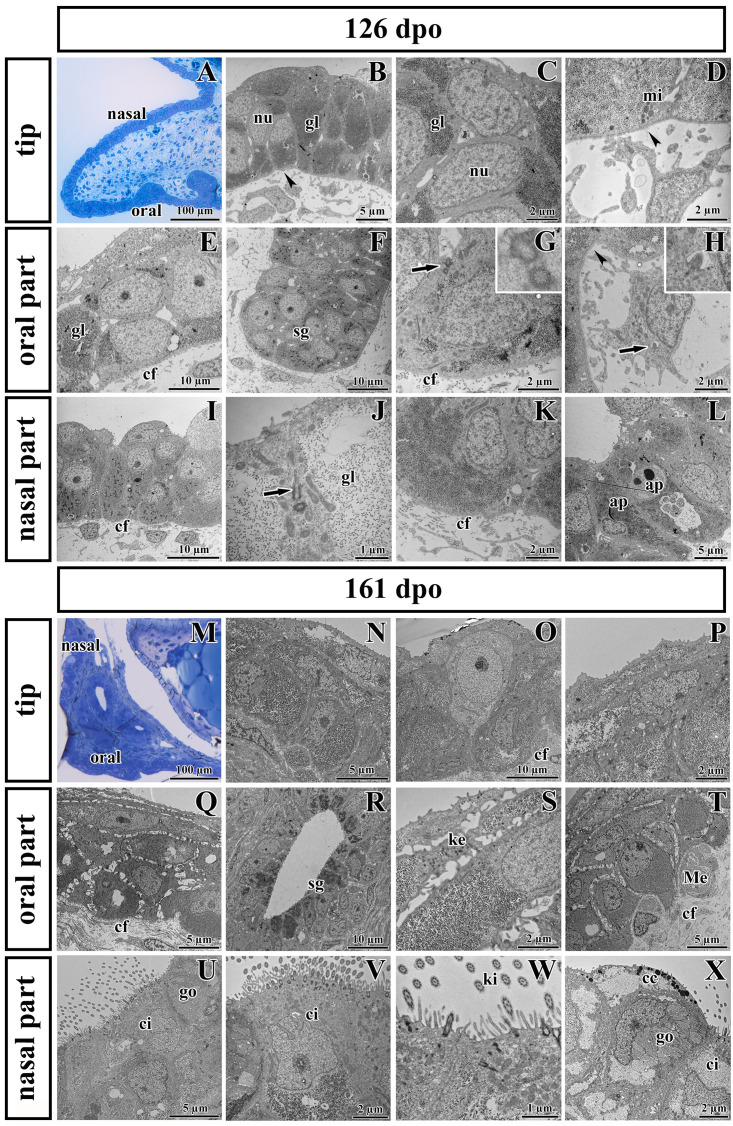
Ultrastructural analysis of the palatal shelves at early and later stages of chameleon pre-hatching development. **(A)** Palatal overview of early developmental stage (126 dpo) stained with Toluidine Blue. **(B–D)** Epithelial cells on the tip of palate with nuclei (nu), high content of glycogen (gl), and mitochondria (mi). **(E–H)** Epithelial (OE) and mesenchymal cells in oral part of the palate. **(F)** The anlage of salivary gland (sg) invaginated from the OE. Primary cilium (arrow) in basal cell of salivary gland. **(H)** Primary cilium in mesenchymal cell (arrow). **(I–L)** Nasal part of palate with club-like cells and cells with high amount of glycogen (gl). **(J)** Primary cilium in epithelial cell (arrow). **(L)** Apoptotic bodies (ap) are present in cytoplasm of epithelial cells close to the nasal cavity. Collagen fibrils (cf) are abundant in mesenchyme with the exception of the tip of palate (arrowheads, **B,D**) and the area surrounding future salivary duct (arrowhead, **H**). **(M)** Palatal overview of later developmental stage (161 dpo) stained with Toluidine Blue. **(N–P)** Epithelial cells on the tip of the palatal shelf with high content of glycogen, collagen fibrils (cf) in mesenchyme present in high manner. **(Q–T)** Oral part of the palatal shelf with large intercellular spaces between epithelial cells (Q). **(R)** Epithelial protrusion of the salivary gland with luminal cell containing secretory granules (sg). **(S)** Keratohyalin granules are visible in the superficial layers of the oral part of epithelium. **(T)** Meissner corpuscles are surrounded by collagen fibrils (cf). **(U–X)** Nasal part of the palatal shelf with goblet cells (go), ciliated cells (ci) lined by motile cilia (kinocilia, ki) and club-like cells (cc).

Noticeable differences in epithelial lining of individual areas were already determined at the earlier analyzed stage (Figures 7A–L). The tip area corresponded to the future side of the attachment between the palatal shelves and nasal septum in the most rostral region (Figure 7A). This edge of the palatal shelves (medial edge) was covered with less differentiated rounded cells that distinctly protruded from the surface (Figure 7B). Numerous glycogen granules were present in all layers of the tip epithelium including in the most superficial layer (Figure 7C). The mesenchyme in the tip of the palatal shelves was almost free of collagen fibrils (Figure 7D), in contrast to other parts of the shelves.

**FIGURE 8:**
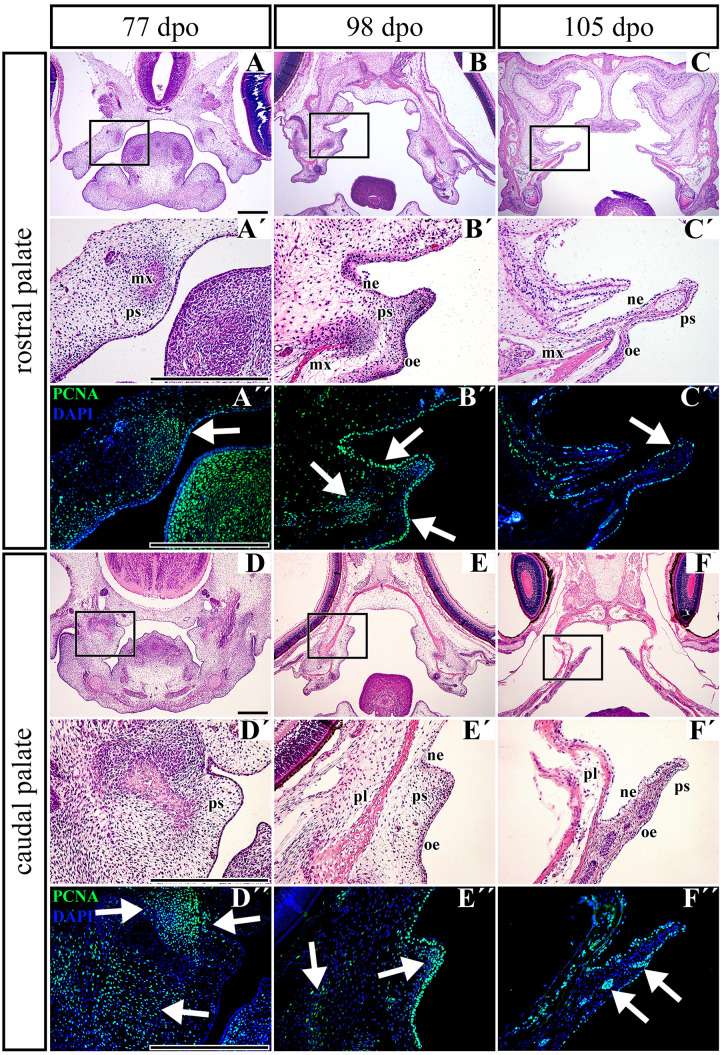
Distribution of proliferating cells in the palatal shelves of chameleon embryos. Cell proliferation in the palatal shelves of 77 dpo **(A–D″)**, 98 dpo **(B–E″)**, and 105 dpo **(C–F″)** stages of the veiled chameleon. Hematoxylin-Eosin staining on the frontal head sections in the lower power and higher power view from the rostral **(A,A′–C,C′)** and caudal **(D,D′–F,F′)** part of the palatal shelves. Immunohistochemical detection of PCNA-positive cells in the higher power view (details from black rectangles) from the rostral **(A″–C″)** and caudal **(D″–F″)** palatal shelves. PCNA-positive cells – green nuclei, PCNA-negative cells – blue nuclei (DAPI). White arrows point regions of PCNA-expressing cells in the forming palatal shelves. dpo, days post oviposition; mx, maxillary bone; ne, nasal epithelium; oe, oral epithelium; pl, palatine bone; ps, palatal shelf. Scale bar: 200 μm.

The epithelial lining of the oral part of the palate (Figures 7E–H) comprised two layers of round or columnar basal cells, and two to four layers of squamous superficial cells covering them. Glycogen granules represented the major substance of the basal cells (Figure 7E), but they were rare in superficial flattened layers, which did not exhibit signs of keratinization. The basal part of the oral epithelium protruded into several epithelial thickenings, which formed primordia of the palatal gland (Figure 7F). Numerous collagen fibrils were found in the mesenchyme surrounding these glandular structures (Figures 7F,G), except of the angle between the oral epithelium and the beginning of future duct of salivary gland (Figure 7H).

The nasal portion of the palatal shelves (Figures 7I–L) exhibited noticeable morphological differences in comparison to the oral epithelium already at this developmental stage (126 dpo). The epithelium was formed by a basal cylindrical layer of cells with high amount of glycogen (Figure 7J). The superficial epithelial cells resembled immature club-like cells, which were covered by numerous microvilli (Figures 7I,J). Numerous collagen fibrils were present directly beneath the basement membrane (Figures 7I,K). Apoptotic bodies were located in the cytoplasm of epithelial cells in the basal angle of the nasal cavity (Figure 7L).

At the later stage, there were even more distinct differences between the oral and nasal parts of the palate (Figures 7M–X). The epithelium differentiated into dissimilar cell types. Squamous multilayered epithelium developed on the oral side of the palatal shelves (Figures 7Q–T) while motile cilia appeared in the nasal part (Figures 7U–X). Superficial cells facing to the oral cavity still did not exhibit signs of keratinization (Figure 7Q). Even at this late stage, the tip of the palatal shelves contained a population of less differentiated cells with reduced intercellular spaces and occasional large light cells penetrating through the epithelium (Figure 7O).

### Proliferation of Mesenchymal Cells Decreases During the Growth of the Palatal Shelves in Pre-hatching Stages of the Veiled Chameleon

Based on the observation that the palatal shelves progressively extend toward the midline but do not reach each other, we wanted to uncover underlying cellular processes. To reveal possible dynamic changes in the proliferation pattern during growth of the palatal shelves, we used proliferating cell nuclear antigen (PCNA) labeling. At the earliest observed stage (77 dpo) in the rostral region (Figures 8A–A″), we detected a large number of PCNA-positive cells in the dorsomedial mesenchyme of the developing maxillary prominence (Figure 8A″, arrow). A large number of proliferating cells was also visible in the adjacent epithelium (Figure 8A″). In the caudal part of the palate (Figures 8D–D″), numerous PCNA-positive cells were located in the mesenchymal condensation of developing bones (Figure 8D″, upper left arrow), and a few PCNA-positive cells were spread in the ventrolateral mesenchyme (Figure 8D″, lower arrow). The epithelium was almost entirely free of proliferating cells; only a few positive cells were detected in the bend of the presumptive nasal epithelium (Figure 8D″, upper right arrow).

During later development (98 dpo) in the rostral area of the developing palatal shelves (Figures 8B–B″), a cluster of PCNA-positive cells was detected in the mesenchymal condensations of the developing palate-forming bones (Figure 8B″, left arrow). The signal was also detected in the oral and nasal epithelium that covered the medial palatal protrusion (Figure 8B″, upper and lower right arrows). In the caudal portion, only a few PCNA-positive cells were detected in the mesenchyme lateral from the mesenchymal condensation (Figure 8E″, left arrow) and in the tip of the palatal shelf (Figure 8E″, middle arrow). On the other hand, the epithelium in the oral and nasal areas was inhabited by a large number of proliferating cells (Figure 8E″).

At the latest observed stage (105 dpo), (Figures 8C–F″), the mesenchyme was almost free of PCNA-positive cells in both the rostral and caudal palatal areas. There were only few proliferating cells located in the oral part of the underlying mesenchyme in the caudal area; they were mostly associated with protruding palatal glands (Figure 8F″, arrows). In contrast, there was still a small amount of PCNA-positive cells equally distributed in the oral and nasal epithelium covering the palatal shelves in both the rostral and caudal areas (Figures 8C″,F″).

### Apoptosis Does Not Significantly Contribute to Palatogenesis in Chameleons

We detected only a small number of apoptotic cells in the palatal shelves during pre-hatching development using the TUNEL assay (Supplementary Figures S3A–F). At early stages, the apoptotic cells were detected in the mesenchymal condensations of the future palate-forming bones (Supplementary Figures S3A″,D″). During later stages, they were located in the mesenchyme surrounding the palatal region bones (Supplementary Figures S3B″,E″) or in the zones, where mesenchymal condensation split between two ossification centers of neighbor membranous bones. However, only a few apoptotic cells were distributed across the epithelium of the oral or nasal part of the palatal shelves or underlying mesenchyme (Supplementary Figures S3C″,F″).

### Polarized SHH Protein Localization in the Mesenchymal Cells of the Palatal Shelves

Epithelial SHH is required for mesenchymal cell proliferation during palatogenesis in mammals (Mo et al., 1997; Zhang et al., 2002). In the mouse, *Shh* mRNA is mainly expressed in the palatal epithelium, while other members of the SHH pathway (the membrane receptors PTCH1 and SMO or the transcription factor GLI1-3) are expressed in the palatal mesenchyme and epithelium. *Shh* expression in the epithelium is reciprocally induced by signals from the mesenchyme and then it signals back to the mesenchyme (Rice et al., 2006).

**FIGURE 9:**
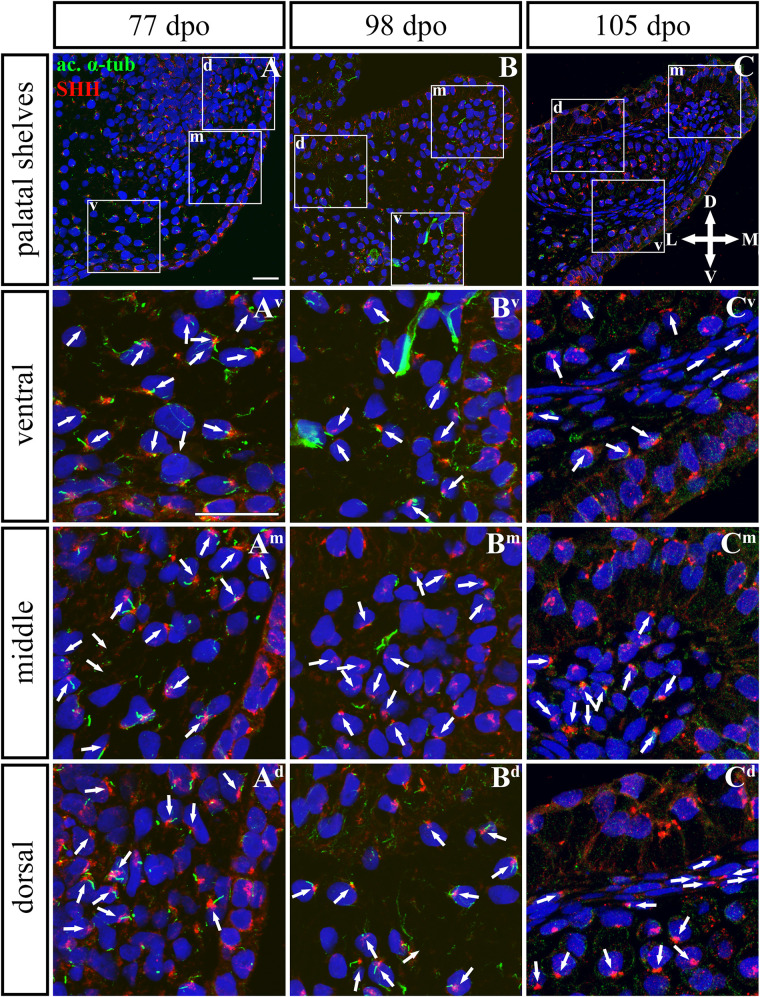
SHH and acetylated α-tubulin protein localization in the rostral palatal shelves in pre-hatching chameleons. Immunodetection of SHH (red) and acetylated α-tubulin (green) proteins on transversal sections. Lower power pictures overview localization at 77 dpo **(A)**, 98 dpo **(B)**, and 105 dpo **(C)** during pre-hatching development. White rectangles define regions focused on ventral (v), middle (m) and dorsal (d) parts of the palatal shelves. Pictures **(A^**v**^–C^**d**^)** show higher power details from ventral, middle and dorsal regions. White arrowheads indicate polarized colocalization of SHH and primary cilium (detected using acetylated α-tubulin) on the same cellular side. Acetylated α-tubulin is present not only in primary cilia, but as well in microtubules of mitotic spindle, therefore there was signal in both structures detected in green. Nuclei are counterstained with DRAQ5. Scale bar: 20 μm.

**FIGURE 10:**
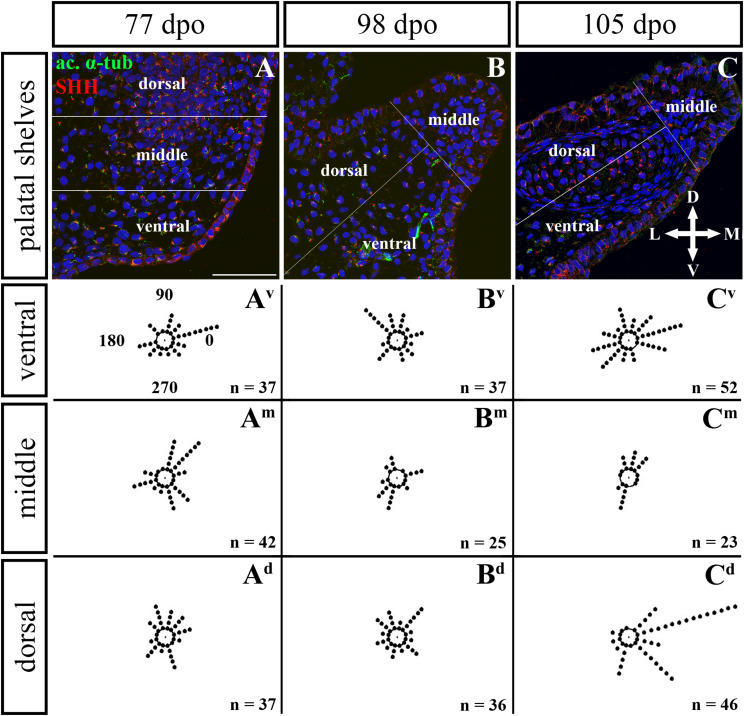
Analysis of SHH ligand polarized localization in the rostral area of the palatal shelves during pre-hatching development. Immunohistochemical detection of SHH polarized localization in mesenchymal cells of the developing palatal shelves. Pictures **(A–C)** show transversal sections divided into ventral, middle and dorsal areas. Rose dot plots of 77 dpo **(A^**v**^,A^**m**^,A^**d**^)**, 98 dpo **(B^**v**^,B^**m**^,B^**d**^)**, and 105 dpo **(C^**v**^,C^**m**^,C^**d**^)** stages show polarity of SHH in individual cells (each dot) according to the dorsoventral and mediolateral axes. The 360 degree circle was divided into 12 zones each with a 30 degree span. 0°, medial direction; 90°, dorsal direction; 180°, lateral direction; 270°, ventral direction. n, number of cells analyzed in each area. Scale bar: 50 μm.

Based on these facts, we asked whether the SHH signal is specifically located in the chameleon palatal shelves in order to stimulate cell proliferation in a specific pattern and direction. During growth of the chameleon palatal shelves, SHH protein was localized in the palatal epithelium and mesenchyme. At the earliest analyzed stage (77 dpo) of the rostral part (Figures 9, 10) of the palatal epithelium, the strongest SHH signal was visible in the medial part of the palatal shelf protrusion (Figures 9A–A^**d**^). Later (98 dpo), SHH was again detected in almost the entire epithelium that covered the developing palatal shelves, but there was apparently less SHH protein compared to the earlier stage (Figures 9B–B^**d**^). However, amount of SHH protein was much reduced in the epithelium, especially in the palatal shelf tip at the oldest analyzed stage (105 dpo) (Figures 9C–C^**d**^). In the rostral palatal mesenchyme at the 77 dpo stage, the SHH signal was located especially in the dorsal mesenchymal condensations (Figures 9A,A^**d**^). Later in development (98 dpo), SHH was spread equally in all the mesenchymal cells (Figure 9B). However, at the 105 dpo stage, while there was some SHH signal in the mesenchymal cells, the amount of SHH protein was strongly reduced (Figure 9C).

**FIGURE 11:**
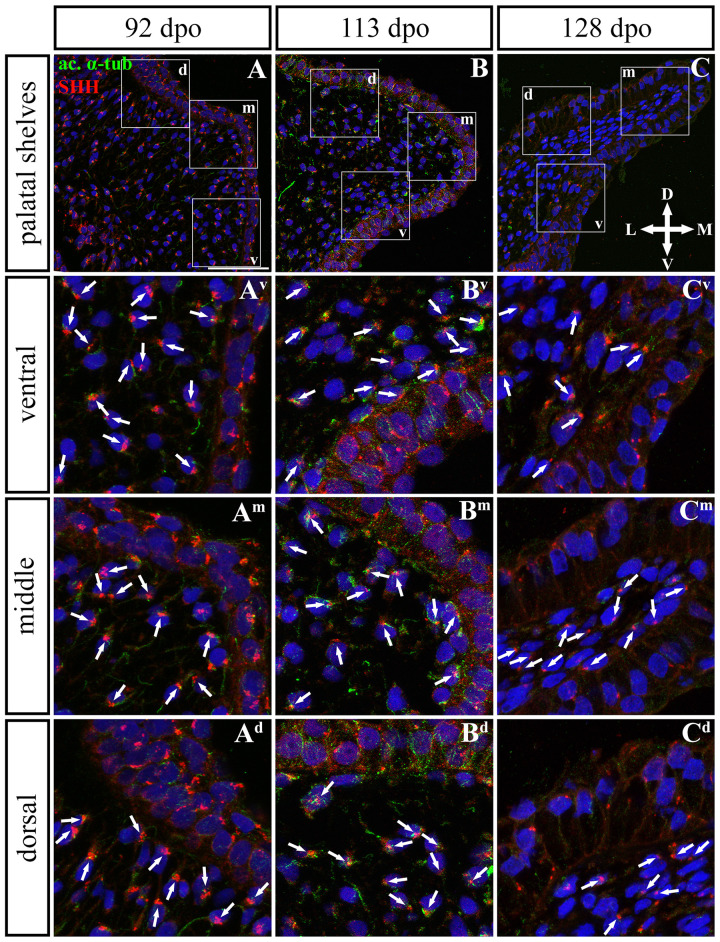
SHH and acetylated α-tubulin protein localization in the caudal palatal shelves in pre-hatching chameleons. Immunodetection of SHH (red) and acetylated α-tubulin (green) proteins on transversal sections. Lower power pictures overview localization at 92 dpo **(A)**, 113 dpo **(B)**, and 128 dpo **(C)** during pre-hatching development. White rectangles define regions focused on ventral (v), middle (m) and dorsal (d) parts of the palatal shelves. Pictures **(A^**v**^–C^**d**^)** show higher power details from ventral, middle and dorsal regions. White arrowheads indicate polarized colocalization of SHH and primary cilium (detected using acetylated α-tubulin) on the same cellular side. Acetylated α-tubulin is present not only in primary cilia, but as well in microtubules of mitotic spindle, therefore there was signal from both detected in green. Nuclei are counterstained with DRAQ5. Scale bar: 20 μm.

**FIGURE 12:**
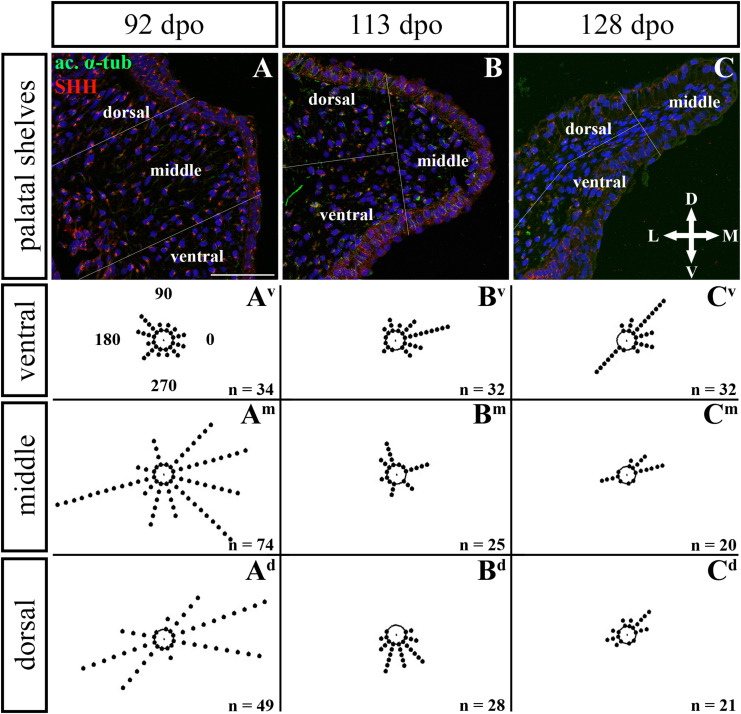
Analysis of SHH ligand polarized localization in the caudal area of the palatal shelves during pre-hatching development. Immunohistochemical detection of SHH polarized localization in mesenchymal cells of the developing palatal shelves. Pictures **(A–C)** show transversal sections divided into ventral, middle and dorsal areas. Rose dot plots of 92 dpo **(A^**v**^,A^**m**^,A^**d**^)**, 113 dpo **(B^**v**^,B^**m**^,B^**d**^)** and 128 dpo **(C^**v**^,C^**m**^,C^**d**^)** stages show polarity of SHH in individual cells (each dot) according to the dorsoventral and mediolateral axes. The 360 degree circle was divided into 12 zones each with a 30 degree span. 0°, medial direction; 90°, dorsal direction; 180°, lateral direction, 270°, ventral direction. n, number of cells analyzed in each area. Scale bar: 50 μm.

In the caudal (Figures 11, 12) epithelium, a strong SHH signal was detected at the early (92 dpo) (Figures 11A–A^**d**^) and middle (113 dpo) (Figures 11B–B^**d**^) analyzed stages. Only few SHH positive cells were detected in the epithelium at the latest observed stage (128 dpo) (Figures 11C–C^**d**^). The SHH localization was similar in the mesenchyme of the caudal and rostral parts of the palatal shelves. SHH was visible in almost all mesenchymal cells at the 92 dpo stage, with the stronger signal detected in the ventrolateral region of the mesenchymal condensation (Figure 11A). At the 113 dpo stage, the SHH signal was slightly decreased (Figure 11B), whereas at the 128 dpo stage, there was only a trace amount of SHH (Figure 9C).

Given that SHH positivity was found in the mesenchymal cells in a specific polarized pattern, we analyzed the distribution of the oriented and localized SHH signal in the mesenchymal cells during outgrowth of the palatal shelves. The palatal shelves at the three developmental stages were divided into three distinct regions (dorsal, middle, and ventral) in both the rostral and caudal areas. SHH signal orientation was analyzed in relation to the position of the nucleus, and rose plots were used to reveal differences in the SHH localization pattern in different areas of the palatal shelves (Figures 10, 12).

Although the analysis at the earliest analyzed stage revealed polarized but more or less random localization of the SHH protein in the mesenchymal cells in both the rostral (77 dpo) (Figures 10A–A^**d**^) and caudal areas (92 dpo) (Figures 12A–A^**d**^), there were some regional exceptions. The analysis in the ventral part of the rostral palatal shelves determined that the most of the cells exhibited SHH localization in medial direction (Figures 10A^**v**^, close to 20^o^ direction). In the caudal palatal shelves, a similar SHH localization pattern was detected in the middle and dorsal parts, where SHH of most of the cells was localized in the dorsomedial and ventromedial direction (Figures 12A^**m**^,A^**d**^, spans directions roughly from 315 to 45°). Moreover, there were many cells with SHH localized in a lateral direction opposite to the medial direction (Figures 12A^**m**^,A^**d**^, close to 200^o^ direction). A similar situation was found in both later stages in the rostral (98 and 105 dpo) (Figures 10B,C) and caudal (113 and 128 dpo) (Figures 12B,C) areas. While in the rostral palatal shelves, polarized localization of the SHH protein was more random (Figures 10B^**v**^–B^**d**^) with respect to the growth direction, in caudal palatal shelves, SHH was localized in ventral mesenchymal cells more medially (Figure 12B^**v**^, close to 20° direction) and in dorsal mesenchymal cells more ventrally (Figure 12B^**d**^, spans directions roughly from 250 to 315°).

At the latest observed stage in both rostral (105 dpo) (Figures 10C–C^**d**^) and caudal (128 dpo) (Figures 12C–C^**d**^) regions, very similar SHH protein localization pattern of the mesenchymal cells was detected in the middle area. With respect to the direction of the most distal part of the palatal shelves, in the rostral region, SHH in most of the cells was localized more in dorsal direction (Figure 10C^**m**^, spans direction from 45 to 90°) and in the caudal region, it was particularly in medial direction (Figure 12C^**m**^, spans direction from 20 to 45°). Most of the dorsal mesenchymal cells in the rostral palatal shelves had the SHH protein localized in the medial direction and this pattern resembled direction of the palatal shelf growth (Figure 10C^**d**^, close to 20° direction). Dorsal mesenchymal cells in the caudal palatal shelves exhibited more random localization of SHH signal (Figure 12C^**d**^). Similarly, random SHH localization was detected in the ventral mesenchymal cells of the rostral palatal shelves (Figure 10C^**v**^), but in the caudal region, ventral mesenchymal cells demonstrated especially polarized localization of SHH (Figure 12C^**v**^) corresponding to the prolonged shape of the palatal shelves in the dorsomedial direction (Figure 12C).

### Colocalization of the Primary Cilia and the SHH Protein in the Mesenchymal Palatal Cells

As the hedgehog signaling was found to be polarized in the mesenchyme and it is well known to be regulated by primary cilia during development, we decided to further follow their appearance in the chameleon palatal shelves. Primary cilia have been observed in a large variety of mammalian cell types or numerous invertebrates (Wheatley et al., 1996; Huangfu et al., 2003; Haycraft et al., 2005; Huangfu and Anderson, 2005; Brugmann et al., 2010; Schock et al., 2016; Hampl et al., 2017), however, they have not yet been described in reptilian species. Therefore, we aimed to analyze the primary cilia structure in chameleon embryos and their possible association with the SHH protein during palatogenesis.

In chameleon embryos, the primary cilia were found in the epithelial and mesenchymal cells of the palatal shelves (Figures 9, 11), including the palatal cartilage (Supplementary Figure S4). Ultrastructural analyses revealed the usual structure of primary cilia in the chameleon palatal shelves. The primary cilia comprised an axoneme that extended from a basal body, which is a modified version of the mother centriole (Supplementary Figure S4) and serves as a microtubule organizing center in the cell. The basal body had a “9 + 0” structure and was composed of nine microtubule triplets that displayed a radial symmetry. The second centriole was arranged orthogonally to the mother centriole. A microtubule-based axoneme consisted of nine doublet microtubules that lacked the central pair of microtubules (9 + 0) and was surrounded by a ciliary membrane (Supplementary Figure S4). In some cases, we observed an irregular arrangement of microtubules in the axoneme (Supplementary Figure S4). In chondrocytes, they were embosomed by numerous membranous structures of Golgi apparatuses and vesicles (Supplementary Figure S4). In epithelial and mesenchymal cells, the vesicles that surrounded the basal body of primary cilia were more random in comparison to chondrocytes (Supplementary Figure S4).

To further evaluate primary cilia distribution in palatal tissues, we used acetylated alpha-tubulin staining to visualize the ciliary axoneme (Figures 10, 12). The primary cilia were associated with the SHH signal but not all SHH-positive cells possessed primary cilia on their surface (Figures 10, 12). This inconsistency was probably associated with the actual cell cycle phase. The primary cilia were more frequent at both earlier observed stages in the rostral area (77 and 98 dpo, Figures 10A,B) as well as caudal area of the palatal shelves (92 and 113 dpo, Figures 12A,B). At the latest observed stages of both analyzed areas (105 dpo, Figure 10C and 128 dpo, Figure 12C), there were only a few primary cilia detected in the palatal shelves.

### *Msx1* and *Meox2* Expression Is Shifted Along the Rostro-Caudal Axis in the Craniofacial Region During Pre-hatching Development of the Veiled Chameleon

Although the molecular regulation of palatogenesis in mammals has been well studied, control of the secondary palate development in non-mammalian species, especially in reptiles, is almost unknown. Therefore, we decided to investigate the molecular mechanisms that contribute to the secondary palate development in chameleon embryos.

For further analyses, we selected three genes (*Msx1*, *Meox2*, and *Pax9*), all of which display a distinct expression pattern during mammalian palatogenesis. In mice, *Msx1* is typically expressed in the rostral part of the maxilla and premaxilla and in the rostral region of the palatal shelves (Zhou et al., 2013). On the contrary, *Meox2* is specific for the caudal zone of the palatal shelves (Jin and Ding, 2006). *Pax9* is expressed in both rostral and caudal parts of the palatal shelves, but the expression increases in the caudal direction (Zhou et al., 2013).

In chameleons, there is a different pattern of membranous bones that form the hard palate in comparison to mammals and other reptiles. While in mammals the secondary palate is formed by palatal processes of the maxilla and only the most caudal region is formed by the palatine bones, in chameleons the maxillary bones are shifted to lateral regions of the upper jaw. Since the main bones of the secondary palate in chameleons are palatines in the rostral region and pterygoids in the caudal region, we hypothesized that there would be a shift in gene expression of region-specific molecules (*Msx1, Meox2*) during chameleon palatogenesis.

**FIGURE 13:**
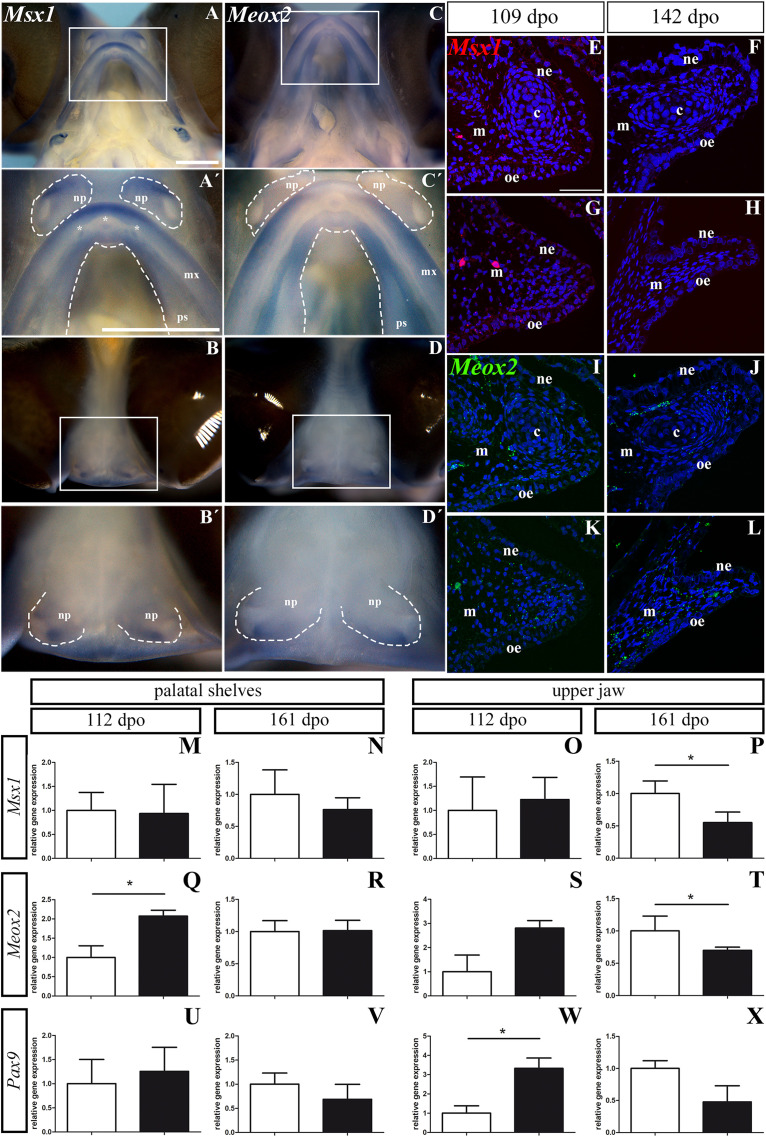
*Msx1, Meox2*, and *Pax9* expression during pre-hatching development of chameleon. Whole mount *in situ* hybridization analysis of *Msx1* and *Meox2* expression on embryo at stage 106 dpo **(A–D′)**. Lower power pictures show overall view on expression of *Msx1* and *Meox2* in either ventral **(A,C)** or frontal **(B,D)** view. Higher power pictures display detail of *Msx1* and *Meox2* expression in the upper jaw region, palatal shelves and nasal pits, from ventral **(A′,C′)** and frontal **(B′,D′)** view. White rectangles define detailed area. The palatal shelves and nasal pits are highlighted with dashed line. Asterisks show expression of *Msx1* in developing teeth. np, nasal pit; mx, maxilla; ps, palatal shelf. Scale bars: 1 mm. RNAScope analysis of *Msx1* and *Meox2* expression **(E–L)**. Transversal sections with focus on the palatal shelves at 109 dpo **(E,G,I,K)** and 142 dpo **(F,H,J,L)** in rostral and caudal regions. Specific expression of *Msx1* in rostral **(E,F)** and caudal **(G,H)** region is shown as red dots. *Meox2* expression in rostral **(I,J)** and in caudal **(K,L)** regions is shown as green dots. Nuclei are counterstained with DAPI. ne, nasal epithelium; oe, oral epithelium; m, mesenchyme; c, cartilage. Scale bar: 50 μm. QPCR analysis of *Msx1, Meox2*, and *Pax9* expression **(M–X)**. Comparison of relative gene expression of *Msx1, Meox2*, and *Pax9* in the palatal shelves at earlier (112 dpo; **M,Q,U**) and later stage (161 dpo; **N,R,V**), and in the upper jaw at 112 dpo **(O,S,W)** and 161 dpo stage **(P,T,X)**, respectively. Gene expression is compared to the rostral region (white columns) and its gene expression level is displayed as value 1.0. The expression in the caudal region (black columns) is displayed as fold change to rostral area. *t*-test; *p* * < 0.05.

First, we used whole-mount ISH to uncover the gene expression pattern of these molecules in the upper jaw and the palatal shelves of chameleon embryos at 106 dpo (Figure 13). Strong expression of *Msx1* was detected in the most rostral zone of the upper jaw (Figure 13A′) as well as in the very most caudal zone of the upper jaw (Figure 13A). It was also expressed very specifically in the developing teeth in the rostral part of the upper jaw (Figure 13A′). Expression of *Msx1* in the palatal shelves was localized only to the verymost rostral zone (Figure 13A′). A positive signal of *Msx1* was also detected around the medial, ventral and dorsal edges of the nasal pits (Figures 13B,B′).

In the upper jaw, *Meox2* was expressed from the rostral to caudal part with the strongest signal detected laterally in the rostral zone (Figures 13C,C′). The very rostral tip of the upper jaw was *Meox2*-negative (Figure 13C′). The whole rostral part of the palatal shelves was *Meox2*-positive, while the caudal region of the palatal shelves was positive only in its lateral part close to the maxilla (Figures 13C,C′). In the nasal pits, we observed a *Meox2* expression pattern that was very similar to that of *Msx1* (Figures 13D,D′), only the signal of *Meox2* was much weaker compared to *Msx1* especially in the ventral view (Figures 13A′,C′).

Next, we wanted to detect expression of the palate-specific genes *Msx1* and *Meox2* in more detail on histological sections. Therefore, we used a fluorescent RNAScope assay for two developmental stages. At the early stage (109 dpo), there was relatively low expression of *Msx1* in the rostral and caudal palatal shelves. While the *Msx1* signal was dispersed within mesenchyme and palatal epithelium in the rostral region (Figure 13E), in the caudal region, there was higher expression in the ventral part of the palatal shelves and especially in the future oral epithelium (Figure 13G). At the later stage (142 dpo), *Msx1* expression was much lower. A weak signal was dispersed in the epithelium and mesenchyme in the rostral region (Figure 13F), but in the caudal region (Figure 13H) only few spots were detectable. *Meox2* was dispersed in the mesenchyme and epithelium of the rostral palatal region (Figure 13I), similar to *Msx1* (Figure 13E) at the earlier observed stage. In the caudal region, *Meox2* was expressed more in the mesenchyme close to the palatal shelf tip, and only a weak signal was detected in the palatal epithelium (Figure 13K). At the later stage, *Meox2* was almost omitted from the epithelium, and also much weaker signal was detected in the mesenchyme in the rostral zone (Figure 13J) compared to the earlier stage (Figure 13I). At the later stage in the caudal region of the palatal shelves, *Meox2* was strongly expressed in the mesenchyme, but the epithelium was almost free of any signal (Figure 13L).

### Analysis of *Msx1, Meox2*, and *Pax9* Expression Levels Confirms the Altered Abundance of the Region-Specific Molecules During Craniofacial Development in Chameleons

To further quantify the observed gene expression pattern changes, we designed chameleon-specific primers for *Msx1*, *Meox2*, and *Pax9* and performed real-time PCR separately in tissues isolated from the rostral and caudal areas of the palatal shelves, as well as from more laterally situated tissues from the upper jaws. During pre-hatching development (112 dpo and 161 dpo) of the veiled chameleon, expression of these three genes varied along the rostro-caudal axis of the both palatal shelves as well as the upper jaws (Figure 13 and Supplementary Figure S5).

In the palatal shelves, the level of *Msx1* expression was highly similar in the rostral (1.0 ± 0.3736) and caudal region (0.93 ± 0.6087, *p* = 0.4494) at the 112 dpo stage (Figure 13M), but at the 161 dpo stage, its expression was reduced in the caudal region (0.7617 ± 0.1860, *p* = 0.2045) relative to the rostral region (Figure 13N). In the upper jaw region, *Msx1* expression was slightly upregulated in the caudal (1.226 ± 0.4593, *p* = 0.3788) compared to the rostral area at 112 dpo (Figure 13O). However, at the 161 dpo stage, the *Msx1* signal was significantly reduced in the caudal region (0.55 ± 0.1629, *p* = 0.0137) compared to the rostral area (Figure 13P).

Significantly higher *Meox2* expression was detected in the caudal region (2.07 ± 0.1522, *p* = 0.0244) of the palatal shelves compared to the rostral region at 112 dpo (Figure 13Q). During later pre-hatching development, *Meox2* levels were comparable in both the rostral (1.00 ± 0.1692) and caudal regions (1.013 ± 0.1617, *p* = 0.4680) of the palatal shelves (Figure 13R). In the upper jaw region, *Meox2* was much highly expressed in the caudal region (2.81 ± 0.3105, *p* = 0.0606) when compared to the rostral area of 112 dpo stage (Figure 13S). Conversely, significantly lower expression was detected in the caudal region (0.69 ± 0.049, *p* = 0.0460) compared to the rostral area at 161 dpo stage (Figure 13T).

In the palatal shelves, *Pax9* expression was higher in the caudal area (1.256 ± 0.4985, *p* = 0.3523) compared to the rostral region at the 112 dpo stage (Figure 13U), but its expression was downregulated in the caudal area (0.687 ± 0.3111, *p* = 0.2111) relative to the rostral area at the 161 dpo stage (Figure 13V). The expression pattern in the upper jaw region was similar to the palatal shelves, with significantly higher expression in the caudal area (3.329 ± 0.5320, *p* = 0.0286) compared to rostral at 112 dpo stage (Figure 13W). Later in development, we again detected downregulated expression of *Pax9* in the caudal area (0.478 ± 0.2512, *p* = 0.0763) compared to the rostral area of the upper jaw region (Figure 13X).

## Discussion

Chameleons are well known for their colorful skin, ability to change skin pigmentation pattern, independently moving eyes, and their prey hunting strategy using a very quick, ballistic, and sticky tongue. Therefore, the veiled chameleon is one of the most frequent bred lizards as a pet. However, the veiled chameleon has recently gained the attention of developmental and experimental biologists. It has already been used to study gastrulation (Stower et al., 2015), neural crest migration (Diaz et al., 2019), limb patterning (Diaz and Trainor, 2015), jaw apparatus morphology (Iordansky, 2016), pigmentation, communication, embryonic diapause, and other aspects, including their development (reviewed in Diaz et al., 2015). Until now, research on a feeding apparatus in chameleons has focused mainly on their tongue (Anderson and Deban, 2010, 2012; Herrel et al., 2014) or teeth (Buchtová et al., 2013; Zahradnicek et al., 2014; Dosedělová et al., 2016). However, other craniofacial structures also exhibit interesting features, and thus we focused on the secondary palate formation, bones that support the secondary palate during both pre- and post-hatching developmental periods, and the mechanism involved in the growth of the palatal shelves. As the veiled chameleon develops large palatal shelves, which can reach the midline during post-hatching stages, it makes this model unique from the EVO-DEVO perspective.

Moreover, it is necessary to mention that the chameleon genome has not been fully sequenced and annotated yet, a factor that makes molecular studies more difficult in comparison to other common model animals. Molecular approaches can be partially compensated by the recently published veiled chameleon transcriptome (Pinto et al., 2019). Another obstacle is the imbalance between developmental stages and difficulties in exact timing of pre-hatching development (Andrews and Donoghue, 2004; Andrews, 2007). This deficit is partly caused by the embryonic diapause, the duration of which can differ between egg clusters, as well as the large variability in the speed of developmental progress among individual embryos in dependence on conditions of external environment such as temperature or humidity during egg incubation.

### Inter- and Intraspecies Morphological Variability of the Palatal Shelves

**FIGURE 14:**
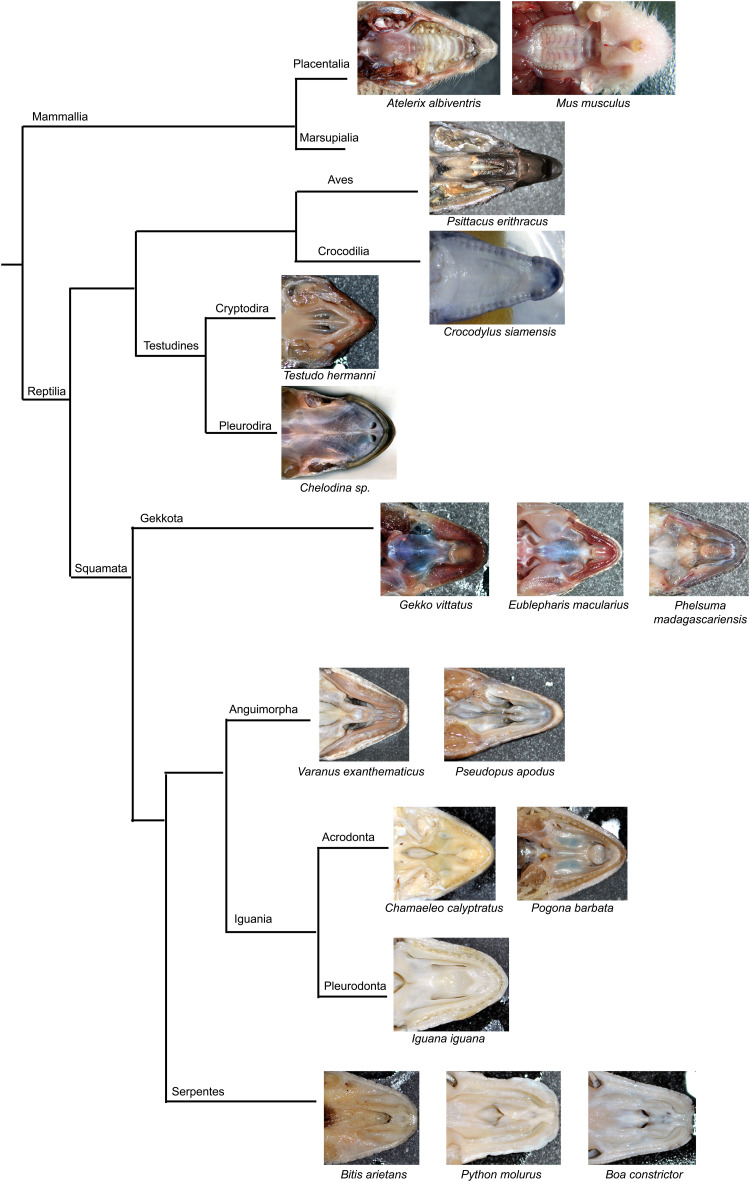
Simplified phylogenetic tree with displayed secondary palate morphology.

The secondary palate, develop in different species with variable degree from small processes located laterally on the maxillae to large palatal shelves meeting in the midline. Crocodilians exhibit typically fused palatal shelves and form an enclosed secondary palate as they live in an aquatic environment and need to completely separate the nasal and oral cavity. Similarly, mammals create the complete secondary palate, but it rather serves as an apparatus for suckling of milk and later in development for verbal communication (Kimmel et al., 2009; Abramyan and Richman, 2015). However, in most reptiles (Figure 14), very small palatal shelves are formed (e.g., geckos, iguana). In other reptiles, e.g., in snakes and turtles, the palatal shelves can be very rudimental with large communication between the oral and nasal cavity (Figure 14). On the other hand, birds develop the palatal shelves largely protruding into the midline, but they do not fuse with the opposite side leaving narrow spacing between them.

Interestingly, the macroscopic morphology of the chameleon palatal shelves varies in post-hatching animals and its appearance is not associated to sex or size of animals. We observed some individuals with large palatal shelves, which were in direct contact in the midline, but most of the animals exhibited a space between the shelves. This intraspecific variation in palate closure among chameleons can be caused by genetic variation but also by environmental causes. As all the juvenile and adult chameleons used in this study were obtained from several breeders and thus bred under different conditions, one of the aspects of the variability observed by us could be different feeding and environmental conditions under which the individuals grew. Other possibility is an influence of genetic variation. Unfortunately, we were not able to evaluate correlation of the palatal shelves expansion and genetic variation in *C. calyptratus*, however, the usage of support vector machine classification approaches, similar as was done for the testing of correlation between male color pattern variation in chameleon and molecular genetic population structure (Grbic et al., 2015) could help to uncover such associations in future.

### Skeletal Bones That Form the Secondary Palate in Reptiles

**FIGURE 15:**
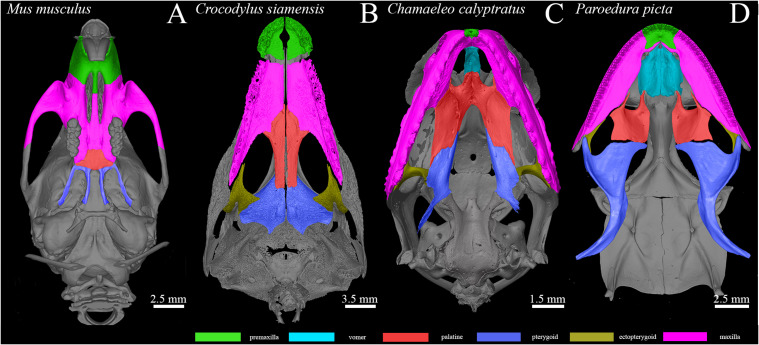
Species-specific arrangement of skeleton contributing to the secondary palate. MicroCT analysis comparing the composition of the palate-forming bones on whole mount cranial skeletons of four different species: *Mus musculus* **(A)**, *Crocodylus siamensis* **(B)**, *Chamaeleo calyptratus* **(C)**, and *Paroedura picta* **(D)**. Individual bones are marked by different colors for better recognition. Each picture has its scale bar with value displayed.

The secondary palate of amniotes is composed of several membranous bones. Their pattern, size of individual skeletal elements, and the extent of their contribution to the secondary palate varies among amniote species (Hanken and Thorogood, 1993). In chameleons, the identity of bones in the palatal area is similar to other higher vertebrates with membranous bones that protrude into the large secondary palate. However, there are significant differences when comparing the pattern of these bones with other species that exhibit the palatal closure. In mammals and crocodilians, the most rostral part of the palate is formed by the premaxilla, and the largest part of the hard palate is supported by medial palatal protrusions of the maxillary bones joined together with a suture at the midline. Paired palatine bones are located caudally from the maxillary bones (Figures 15A,B). This location contrasts with chameleons, where the premaxilla comprises only a small proportion of the most rostral zone of the upper jaw. The maxilla is located generally laterally in the jaw and is the main tooth bearing bone (Figure 15C). The largest proportion of the chameleon palatal shelves is formed by the palatine bones expanding into their rostral area. In the caudal palatal area, the skeletal pattern in chameleons is similar to crocodilians, with a large and flattened pterygoid body. The pterygoid is also extensive and contributes to the secondary palate in fresh water turtles (Abramyan et al., 2014) and sea turtles (Jones et al., 2012). In contrast, mammalian pterygoid bones are reduced caudally either to small bones, e.g., in opossum (Mohamed, 2018), or as ventral processes of the sphenoid bone (pterygoid hamulus) in humans. These bones contribute to a proper function of the soft palate (Krmpotić-Nemanić et al., 2006).

A more comparable pattern of the palate-forming bones to chameleon can be observed in reptiles with open secondary palates, e.g., the geckos (Figure 15D). The premaxilla, maxilla, and vomer exhibit almost the same arrangement compared to chameleon. Similar to the chameleon, paired palatine bones are located in the middle of the palatal shelves: rostrally they are connected to the vomer, laterally to the maxilla and ectopterygoid, and caudally to the pterygoid (Figure 15D). The maxillary bones are shorter in the gecko and located more rostrally compared to the chameleon. The largest bone of the gecko skull, the pterygoid, is located caudally from the palatine bones (Daza et al., 2015). It was previously proposed that changes in pterygoid size and shape may be associated with differences in jaw movement during food processing or mastication (Crompton, 1995).

The caudal part of the chameleon palate is supported by the ectopterygoid that connects the maxillary, jugal, and pterygoid bones. The presence of the ectopterygoid in mammals is still controversial; it is most often considered to be a part of the pterygoid bone or pterygoid hamulus (Presley and Steel, 1978). In reptiles, however, the ectopterygoid represents an important bone that links the outer and inner rows of the upper jaw skeletal elements. The analysis of wall thickness also revealed that the ectopterygoid has one of the thickest bony trabecula in chameleon. This observation supports the idea of the ectopterygoid as the main bone that carries the pressure load.

### Cartilaginous Structures Can Contribute to the Secondary Palate Formation

During ontogenetic development, the hard palate of birds and mammals is supported by membranous bones, but in the chameleon, we also observed a cartilaginous structure that invaded the palatal shelves. Interestingly, the cartilage protrudes into the palatal shelves also in mammals during their suckling period (Li et al., 2016). In this group, the palatal cartilage was described as a newly formed element that wholly develops in a membranous bone-forming tissue. In chameleons, the palatal cartilage develops as a part and protrusion of the chondrocranium. It separates from the nasal septum cartilage in the rostral palatal region, and its most caudal portion can reach almost the middle of the palatine bone. This cartilage is located in the palatal region from early pre-hatching development, through juvenile stages into adulthood. Although development of the chondrocranium was well described in several reptilian species (Hernández-Jaimes et al., 2012; Diaz and Trainor, 2019), to our knowledge, a similar structure for this palatal cartilage has not been described in other species.

### The Main Palate-Forming Bones Are the First Bones to Ossify in the Craniofacial Skeleton in Species With an Enclosed Secondary Palate

Ossification centers of the craniofacial bones first appear in human around 6–7 weeks (40–42 days) post-conception in the developing mandibular, maxillary, and premaxillary bones. A week later (day 57), the vomer, palatine bones, pterygoid plates of the sphenoid bones, and zygomatic bones appear. Ossification centers of other craniofacial bones appear also during week 8 post-conception. Based on this information (Sperber et al., 2012), the main palate-forming bones – maxillary and premaxillary – are the first bones of the craniofacial skeleton to ossify in humans. Furthermore, in embryos of smaller mammals, such as the house mouse (*Mus musculus*) or golden hamster (*Mesocricetus auratus*), ossification centers for all the bones, that contribute to form the hard palate, appear during the initial phase of ossification: day 15 post-conception in mice (Johnson, 1933) and day 12 in hamsters (Kanazawa and Mochizuki, 1974).

The developmental sequences are similar in reptiles; ossification of the palate-forming bones is initiated first, but the order of the ossification of individual elements differs depending on their contribution to the palate. In the American alligator (*Alligator mississippiensis*), the first ossification is visible in the forming pterygoid and maxillary bones at 26 days post-oviposition (dpo). At 28 dpo, the ossification centers of premaxillary and jugal bones appear. At 35 dpo, the ectopterygoid, palatine, and vomer ossification centers are visible (Rieppel, 1993). In the bearded dragon lizard (*Pogona vitticeps*), the first ossification center appears in the forming pterygoid bone at 18 dpo, followed by the palatine, maxillary, and jugal bones at 24 dpo. Finally, at 28 dpo, the ossification centers emerge in the premaxillary, vomer, and ectopterygoid bones (Ollonen et al., 2018). In the Andean lizard (*Ptychoglossus bicolor*), the ossification timing is similar: the first ossification centers appear in the developing pterygoid, maxillary, jugal and prefrontal bones at 35 dpo, and the premaxillary, vomer and ectopterygoid centers are visible at 39 dpo (Hernández-Jaimes et al., 2012). Even in turtles, where the arrangement of the skeletal elements is altered in comparison to squamate reptiles, such as the alligator snapping turtle (*Macrochelys temminckii*) representing freshwater turtles, the first ossification center appears in the maxilla at stage 17. The pterygoid ossification center is visible at stage 18, while the centers are visible at stage 20 for the premaxilla and at stage 21 for the vomer, palatine and jugal bones (Sheil, 2005). In this study, we determined, that the first bones ossified in the palatal region of the veiled chameleon were the pterygoid and palatine bones. Slightly later, ossification centers of the jugal bones appeared, followed by the maxillary and ectopterygoid bones. This sequential ossification is very similar to the bearded dragon lizard (*P. vitticeps*) with the only exception of timing of the maxillary bone ossification. The last bones ossified in the palatal region of chameleons are the vomer and premaxillary bones. Based on these few examples of reptilian and non-reptilian species, it is clear that the ossification of the palate-forming skeletal elements starts earlier during pre-hatching development to support developing palatal structures.

### Outgrowth of the Palatal Shelves and Their Directionality

In mammals, medial bulge-like protrusions are formed on the lateral maxillary prominences at the beginning of the secondary palate development. These protrusions later grow and transform into the prolonged palatal shelves. First, the palatal shelves protrude vertically downward alongside the tongue and then they elongate in the horizontal direction. After reorientation into the horizontal plane, the palatal shelves grow toward the midline, where they finally fuse. The outgrowth of the palatal shelves from the paired maxillary prominences is a typical feature of amniote craniofacial development (Tamarin, 1982; Bush and Jiang, 2012). This phenomenon contrasts with more basal vertebrates, where the palatal shelves do not develop and the choanae open into the oral cavity (Jankowski, 2013). In amniotes, there is great variability in the intensity of the palatal shelves outgrowth or directionality of their initial outgrowth. In most lizards, snakes, and birds, the palatal shelves initially grow horizontally without fusion, and the spacing between them remains visible (Kimmel et al., 2009). In crocodilians, the palatal shelves expand also in the horizontal direction from the beginning of their development (Ferguson, 1981b). However, in the most caudal part of the palate, the shelves first protrude vertically and then they turn into horizontal position (Ferguson, 1981a,b,^1987^), which is more similar to the mammalian developmental pattern, where the palatal shelves also first expand vertically and later turn horizontally (Bush and Jiang, 2012). In mammals, there are also differences in the morphology and outgrowth of the palatal shelves in the anterior and posterior areas of the secondary palate. In the anterior part, the shelves exhibit a finger-like shape that protrudes into the oral cavity. In the middle palatal zone, the palatal shelves have a more triangular shape while caudal parts have a rounded distal end (Bush and Jiang, 2012).

In this study, we revealed that the palatal shelves of chameleons do not grow strictly in the horizontal direction. They rather extend dorsomedially from the beginning of their initiation. Furthermore, these animals possess an open fissure between the palatal shelves when they hatch. However, the jaw and palate extensively develop and continue to grow even during post-hatching development. In fact, the palatal shelves directly contact in the midline of some adult animals. Certain rostro-caudal differences in the direction of the palatal shelves growth were observed already at the earliest analyzed embryonic stage. While in the rostral palatal region, there was medial round thickening of the future palatal shelf, in the caudal region, there was already a finger-like projection oriented into the horizontal plane. This shape resembles the palatal shelves of the caudal region at E14 in mice (Yu and Ornitz, 2011).

### Specific Distribution of Proliferating Cells and Cellular Polarity Is Associated With the Directionality of the Palatal Shelves Outgrowth

The outgrowth of the palatal shelves is characterized by localized proliferation during early stages of development. As the result of this process, we observed differential outgrowth on one side of the palatal shelf. This conserved developmental mechanism was previously described for mammalian species (Iwabe et al., 2005), bird and reptilian (Abramyan et al., 2014) embryos. On the other hand, the loss of proliferation in certain areas was proposed to be the main cause of the palatal shelf outgrowth failure in turtles (Abramyan et al., 2014). In chameleons, we observed an unequal distribution of proliferating cells in the palatal shelves of pre-hatching embryos, with higher proliferation in the dorsal part of the maxillary protrusion resulting in formation of the large palatal shelves. On the other hand, as the animal aged, there was an apparent decrease in the number of proliferating cells, especially in the mesenchyme. This phenomenon seems to contribute to slowing down their outgrowth as the level of proliferation during critical early developmental stages is not effective enough in the palatal shelves for their reaching the midline even though their slow growth continues during post-hatching stages. Therefore, most adult chameleons have a gap between the collateral palatal shelves in the midline and the secondary palate thus remains open.

SHH and bone morphogenetic protein (BMP) signaling are key molecular pathways during the palatal shelves outgrowth (Zhang et al., 2002; Rice et al., 2006). In mammals, SHH expression is restricted to the lateral epithelium, especially to form the rugae palatinae. In chameleons, a stronger SHH signal was observed in both the epithelium and mesenchyme at early stages of development. During pre-hatching development, the amount of SHH protein visibly decreased in the mesenchyme and oral epithelium of the palatal shelves. This observed decrease of the SHH signal corresponded with decreased proliferation, which was followed by reduced growth of the palatal shelves in the dorsomedial direction in chameleons.

From the above mentioned observations, two questions emerge: Why do the palatal shelves not grow first vertically and then reorient to the horizontal direction in chameleons like in mammals? On the other hand, why do they not grow horizontally like in crocodilians, but rather extend in the dorsomedial direction toward each other and the nasal septum? It was proposed that the growth direction of the palatal shelves during development is influenced by the presence of a large tongue, which functions as a physical barrier. During early mammalian development, the tongue fills almost the entire oronasal cavity, and thus there is no space for the palatal shelves to grow horizontally, and they have to extend first vertically along the tongue. Later, when the head grows along the dorsoventral axis, there is more space above the large tongue. At that time, the palatal shelves can reorient to the horizontal position and grow toward each other to form the complete palate. On the other hand, in crocodilians the tongue is more flattened, so there is no barrier that would impede direct horizontal growth of the palatal shelves. In line with this proposed hypothesis, the chameleon development lies somewhere between mammals and crocodilians. There is a relatively massive tongue in the oronasal cavity, but there is still some space left above it. This state is very similar to the late stage of mammalian palatogenesis during reorientation of the palatal shelves from a vertical to a horizontal position, when there is already free space for their horizontal growth. However, a slight developmental difference which we should mention is that the chameleon palatal shelves grow all along the jaw in dorsomedial direction in order to overgrow the tongue.

Another aspect of the palatal shelves’ growth directionality could be polarized localization of the SHH protein and the localization of the primary cilia on the palatal mesenchymal cells. It has been previously reported during development of zebrafish craniofacial cartilages, that mesenchymal cells are oriented to the center of condensations based on expression of anti-gamma tubulin (labeling microtubule organizing center), which colocalized with the primary cilia (Le Pabic et al., 2014). The direction of the chameleon palatal shelves growth corresponds with the cellular localization of SHH protein and the primary cilia in several palatal regions during pre-hatching development, but not all of them. Based on our observations, specific SHH localization seems to be a consequence of mesenchymal cells polarization as SHH protein is bounded here to Hh receptors, which are enriched in the primary cilia and in the surrounding cellular membrane. Therefore, a combination of polarized localization of the primary cilia and associated SHH localization, and directed proliferation could be one of the causes resulting in outgrowth of the palatal shelves typical for pre-hatching chameleons. However, this phenomenon will be necessary to test experimentally in future.

On the other hand, it is necessary to mention, that alteration of the cell dynamics or cell polarity do not need to be only processes contributing to the palatal shelves’ growth. The growth of bones and larger scale craniofacial architectural changes affect reciprocal position of the palatal shelves. As the skeletal architecture changes with age, the snout elongates, and the palatal shelves can be translocated to each other by their passive movement associated with narrowing of the midfacial structures. Such changes are common in embryonic and post embryonic ontogeny in reptiles, especially in chameleons that are known for their midline reduction (Rieppel and Crumly, 2009; Diaz and Trainor, 2019).

### Expression of *Msx1*, *Meox2*, and *Pax9* During Palate Development in Non-mammalian Species

Palatogenesis is a highly regulated morphogenetic process; the speed and direction of outgrowths from the maxillary prominences must be precisely controlled. The complexity of the palatogenesis control is reflected by the common occurrence of a cleft palate in humans. While the regulation of mammalian palatogenesis has been well studied, knowledge about the genetic control of the secondary palate development in non-mammalian species, especially in reptiles, has been almost entirely omitted.

*Msx1, Meox2, and Pax9* genes display distinct expression patterns during mammalian palatogenesis and mutations in these genes cause developmental defects, typically a cleft palate. In mouse, *Msx1* is typically expressed in the rostral palatal shelves (Zhou et al., 2013) while *Meox2* is rather expressed in the caudal palatal shelves (Jin and Ding, 2006). *Pax9* expression increases in the caudal direction in the palatal shelves (Zhou et al., 2013). An expression pattern similar to mouse embryos was observed in chicken with *Msx1* and *Pax9* genes expressed during early development in the maxillary prominences (Namkoong, 2015).

In chameleon, we detected expression of *Msx1* reduced in caudal parts of the palatal shelves and the upper jaw at the 161 dpo stage. Similarly in the chicken model at the HH26 stage, *Msx1* expression is also limited to the rostral part of the maxillary prominence (Namkoong, 2015). During embryonic development (stages 13, 15, and 17) of the Siamese crocodile (*Crocodylus siamensis*) and Chinese softshell turtle (*Pelodiscus sinensis*), *Msx1* expression was detected in the forming palate and maxillary prominences only at the stage 17 (rostro-caudal comparison not shown) (Tokita et al., 2013).

Here, we determined that expression pattern of *Meox2* in chameleon changes during embryonic development in the palatal shelves and the upper jaw region. While at an early stage (112 dpo), *Meox2* expression was much higher in the caudal areas, at later stage (161 dpo), its expression was the same in both regions of the palatal shelves and even higher in the rostral area of the upper jaw region. This pattern is similar to the expression pattern in mouse embryos (Jin and Ding, 2015).

At the early stage (112 dpo) in chameleon, *Pax9* expression was higher in the caudal region of the both palatal shelves and upper jaw region, which is in concert with the expression pattern in mouse embryos (Zhou et al., 2013). The same *Pax9* expression pattern (levels are higher in caudal region of the maxillary prominence) was shown in chicken at the HH24 stage (Namkoong, 2015). Conversely, in chameleon at the 161 dpo stage, *Pax9* was highly expressed in the rostral regions of the both palatal shelves and upper jaw region. In the Siamese crocodile, *Pax9* expression was detected in all three analyzed embryonic stages within forming upper jaw and surrounding tissues, with the strongest expression visible in the forming palatal shelves at stage 17. In the Chinese softshell turtle, *Pax9* was detected in the medial part of the maxillary prominences at stage 13, in the forming palatal shelves at stage 15, and in the medial part of the upper jaw at stage 17 (Tokita et al., 2013).

In this study, we found that all these analyzed genes were expressed during palate development also in a non-mammalian model, the veiled chameleon. Their expression levels differ along the rostro-caudal axis of the palatal shelves during embryonic development. While expression of the *Msx1* and *Pax9* genes was demonstrated during craniofacial embryonic development also in other reptiles, the Siamese crocodile and Chinese softshell turtle (Tokita et al., 2013), unfortunately, their analyzes was not focused on palatogenesis and the level of their expression could not be correlated with the rostro-caudal differences in the palatal shelves morphogenesis, which will be necessary to follow for possible comparisons in the future.

### Primary Cilia in Non-mammalian Models

The primary cilia are essential cellular structures that are required for a proper function of several signaling pathways. Non-motile cilia should be present on almost all mammalian cells; they have also been detected in several non-mammalian species. The primary cilia were detected in the chicken, which is used as a model organism for human craniofacial ciliopathies (Schock et al., 2016). Furthermore, zebrafish have been used to model human ciliopathies with a craniofacial phenotype and defective SHH signaling (Duldulao et al., 2009). In *Xenopus*, primary cilia are important structures for signaling pathways and embryonic development (Shi et al., 2014). Although reptiles are becoming more popular among other classic model organism, there is no clear information about analysis or detection of the primary cilia in reptiles. This study is the first to display the primary cilia morphology in reptiles. Interestingly, we found an association of SHH polarity with the presence of the primary cilia in the palatal mesenchymal cells, which correlated with the growth direction of the palatal shelves of the veiled chameleon. However, the real significance of this association will require functional tests.

## Conclusion

In conclusion, our results revealed several specific morphological features of the secondary palate formation in chameleons. However, there are some remaining developmental questions: Why are the palatal shelves so well developed in chameleons and why does the process of oral and nasal cavity separation continue during post-hatching development up to the complete closure of the palatal shelves in some adult chameleons? Based on the observed heterogenous morphology of the secondary palate in adult individuals, it is not clear if there is any functional advantage for the animals with separated cavities. One possibility is a mechanical need for closing the secondary palate to enable an effective tongue catapult to precisely control its direction. Moreover, the large palatal shelves can be developed just to keep food out of the nasal cavity, similarly to what has been described for birds (Jankowski, 2013). These functional causes of the secondary palate development in chameleons are not known and it will be interesting to uncover them in the future.

## Data Availability Statement

The raw data supporting the conclusions of this article will be made available by the authors, without undue reservation, to any qualified researcher.

## Ethics Statement

All procedures were conducted following a protocol approved by the Laboratory Animal Science Committee of the Institute of Animal Physiology and Genetics, Academy of Sciences (licence no. CZ21760006, Liběchov, Czech Republic) under supervision of the Regional Veterinary Administration (Ústecký Region).

## Author Contributions

MH, JD, MK, HD, AB, OZ, MP, MM, and TZ performed the analysis. OZ, MP, and JK provided the material. MH, JD, MK, MM, JK, and MB drafted the manuscript. All authors contributed to the article writing and approved the submitted version.

## Conflict of Interest

The authors declare that the research was conducted in the absence of any commercial or financial relationships that could be construed as a potential conflict of interest.

## Supplementary Material

MATERIAL S1 Chameleon sequences used for primer and probe design.

FIGURE S1 Detailed palatal and lateral view on chameleon skull at pre-hatching stages. Palatal view on the chameleon head at earlier stage (98 dpo) and at later stage (112 dpo) **(A,B)**. Lateral view on the embryo at 98 dpo stage demonstrate ossification centers of maxillary bone (mx), jugal bone (j), and post-orbitofrontal (pfo) bone **(A′,A″)**. Lateral view on the embryo of the 112 dpo old with focus ossification centers of maxillary bone (mx), jugal bone (j), and post-orbitofrontal (pfo) bone **(B′)**. Pictures orientation: left (caudal, C), right (rostral, R). Scale bars: 1 mm.

FIGURE S2 Palatal shelves morphology and surface structures arrangement of chameleon embryos in scanning electron microscope. Palatal view on prehatching stages of chameleon at age 17 weeks (weight of embryo 0.36 g), **(A–E)**, at age 18 weeks (weight of embryo 0.42 g), **(F–J)** and at age 20 weeks (weight of embryo 0.53 g) **(K–O)**. Low power view on the palatal shelves (ps) **(A,F,K)**. Higher magnification on the rostral areas of the palatal shelves with magnification on the palatal edge (pe), **(B,C,G,H,L,M)**. Higher magnification on the caudal areas of the palatal shelves with magnification on surface structures highlighting motile cilia in the oldest embryo (yellow arrow), **(D,E,I,J,N,O)**. Scale bars: **(A,F,K)** – 2 mm, **(B,G,L)** – 200 μm, **(C,H,M)** – 10 μm, **(D,I,N)** – 200 μm, **(E,J,O)** – 10 μm.

FIGURE S3 Distribution of apoptotic cells in chameleon embryos. Cell death in the palatal shelves of three different pre-hatching stages of the veiled chameleon. HE stained frontal head sections in the lower power from rostral **(A–C)** and caudal **(D–F)** areas of the palatal shelves. Higher power view (details from black rectangles) of TUNEL-positive cells on transversal sections through head in rostral **(A′–C′)** and caudal **(D′–F′)** areas of the palatal shelves. Details of the palatal shelves with black arrows pointing on TUNEL-positive cells (brown) in either rostral **(A″–C″)** or caudal **(D″–F″)** regions of the palatal shelves. Nuclei (blue) are counterstained with Hematoxylin. Ps, palatal shelf. Scale bars: 200 μm, details: 100 μm.

FIGURE S4 Ultrastructure of the palatal cartilage and primary cilia morphology in chameleon embryos. The rudiment of the palatal cartilage was present closely adjacent to nasal part of the palatal shelf and chondroblasts expanded into tip of the palatal shelf. Chondroblasts located on the cartilaginous periphery were flattened, centrally situated cells were oval or round-shaped. Primary cilia were frequently observed in the epithelium, mesenchyme or in the palatal cartilage (arrowheads). In chondrocytes, they were embosomed by membranous structuresof Golgi apparatuses. Bb, basal body; cf, collagen fibrils type II; cp, ciliary pocket; ch, chondroblast; ecm, extracellular matrix; ga, Golgi apparatus; gl, glycogen; pe, perichondrium; re, rough endoplasmic reticulum.

FIGURE S5 Gene expression analyses. Labeled areas from which tissues were collected for QPCR analyses **(A)**. Comparison of gene expression of *Msx1*, *Meox2*, and *Pax9* in the palatal shelves and in the upper jaw during pre-hatching development of the veiled chameleon. Gene expression comparison between individual genes in the palatal shelves of the earlier stage **(B)** and later stage **(C)**, and in the upper jaw of the earlier stage **(D)** and later stage **(E)**. Gene expression is shown as relative gene expression. In each group, gene expression of *Msx1* in the rostral tissue was used as a control for comparison.

TABLE S1 List of contigs used for the primer and probe synthesis.

TABLE S2 List of the primers for PCR and QPCR.
