# Supplementary material for: Polarized Sonic Hedgehog Protein Localization and a Shift in the Expression of Region-Specific Molecules Is Associated With the Secondary Palate Development in the Veiled Chameleon
Source: Front Cell Dev Biol. 2020 Jul 28;8:572. doi: 10.3389/fcell.2020.00572 (PMC7399257; doi:10.3389/fcell.2020.00572)
Supplement: MATERIAL S1 — Chameleon sequences used for primer and probe design. [file Data_Sheet_1.PDF]

Supplementary material S1: Chameleon sequences used for primer and probe design

MEOX2

>D1_embryo_assembly_TRINITY_DN59016_c3_g5_i2_Swissprot_sp|P50222|MEOX2_HUMAN

GCTGCCGCTGCCTCGGCCCGGCACAGCCTGTGCCTCCAGCCCGAGGCGGGCGGGG
CGGCGGAGCTGGGCAGCGGCCCCCCGGGACTGTGCTCCAACAGCGCCTCCAGCTT
GGGCGGCGGAACCGCGACGACGACGGGGCCGGCGTGCGCCCCGGGAGATTACGG
CCGCCAGGCGCTCTCCCCCGCCGAGACCGAGAAGCGGAGCGCCGGCAAGCGGAA
AAGCGACAGCTCAGATTCTCAAGAAGGAAATTATAAATCTGAAGTCAACAGTAA
ACCAAGGAAAGAAAGGACGGCTTTCACCAAAGAGCAGATCAGAGAGCTAGAAG
CAGAATTTGCCCACCACAACTACCTCACCAGGCTGCGGAGATATGAGATAGCAGT
AAACCTGGATCTTACTGAAAGACAGGTAAAAGTTTGGTTCCAGAACCGACGGAT
GAAATGGAAGCGGGTAAAAGGAGGGCAGCAAGGAGCTGCAGCCCGAGAAAAGG
AACTGGTGAACGTGAAAAAGGGCACATTGCTCCCATCTGAGCTTTCCGGGATCGG
CGGCAGCAGCCTCCAGCACACGGGGGACTCACTAGCAAATGACGACAGCCATGA
GAGTGACCACAGCTCCGAGCATGCGCACTTATGATAACACAGGAAGGAATCACA
GCTCTCTTCTCAGGAAAGACATAGTGATGCAGCAAAGCCTTATGTGGACATCCTT
AATGTATGCAAAGGACTTTCTGGCAATGAAAAATAGCTTTGGACTTCCACTCTCT
GCTACTTTTGAATTGTCTTTTTAACACACATTCTGTAAGTGCCCACACCTTAAACG
TGCCTTGCAAAGGGGGAGCAAAGGGCAAGGTTGTTTTTGAACATTCCAGTTTTGT
CTACCATATCAGTGACAACAGGCAAATATTATTTTTGCTTTTGCCATTTCTCTCAA
TAAATGTAAAGTGGTGGGGAGGGG

PAX9

>D1_tissue_assembly_TRINITY_DN47668_c3_g2_i9_Swissprot_sp|Q2VL51|PAX9_LEPED

AAGTAAGTAAGTAAGTAAGTAAGTAAGTAAGTAAGGAAGGAAGGAAGGAAGGA
ATGAACAAACAAATGAATGGGAAAGGAAAGAAAGAACCTAAAAACTCTGTCTGC
TCCTACTGATTGCATCTCATGATGACTATTCTCAGTTCCACATCTGACATGGCTAA
ATGATTGCAGTAGAAAAAATGTTTTGGAAAGTTTCCCAGGAAGTATTTGTATGAC
ATCACATATTATTTTCTTTGCACAGCCTTAGAAATCTACCTTTGTTGGCATTGTAA
ATTAAAAGCATGAAATTCTCATTAAAAAAGCAGCCTCTTATCTTGCTGTCCCAGC
AGTACGGGGTAATATGGCTAGGAAGAAACAGAGATAATTTTATTAAAGCAGTCTT
TCAAAGTCTGTATTGAATGGTGTATTTTTACATTATTGCACTAGACTTACAAAAAT
ACATAATATATTAATATCTTATAATGCTAAGAAATATGTCAATTTTCCATTTTCTT
TCTTTTCATGGTTATTCTTCAACACGGCATCTGCAAGCTTGAGAAGGCTTTCTGAA
ATTATATTCACAACTTTATTATTGTCCAAGTAGAACAGGCCATCTTGAAAAGATT
ATACTGTTAATGCCAAAGGGTAAAAGAGAGCAATCAGAATCCTGGTGGCCACAA
AATGATGCTTGGACAAGAATACAAAAATGGCAGGGAAGGGGGGAGTTTAAAAAG
ATTTTTTTTAACATTATAAATAATATTTGTAGCACCACCACCAATATGCCTAGGAG
TGAAACATGCTTTTGTACAATATTGTAAACTTTACCTAAAGTTTCCTTCTTTTTTAC
ATTCAACAAAACAGGAATTTTAGACATGCTTTCTTCTTTTGTTGTATGCTTGCATG
GTTAAAAAAAACAATATAATGTGGGGTCAGGAGTCTCTGAACACAGATTAGCCA
ACACATTACAGTACAGTTTTCTTTCACAGTGGTTTGGCTAATGCTGGGCATCGTTC

ACCTGCCTCTCTCATGCACAGCCACTGCAACCAGGGGTGTGAGTGTGCCCATAGG
TTCTGCCATGCTTGGGCAGAAGAACTTCCTGGTGGGATTCTGCCCTTGGCTAATTA
AAAAAAACACAAAATTCACATTAAACATAAGCCTCCACTTTGTTCAGATTCTTTC
TATCCATTGCATAGCTGTCAAGTATCATGAATTAGCTGGGATTTCCCATATTTTGG
GGCTCCTTCCCTCTTTTTTCTGCTGCCATCTCATATTTTGGCAGCTGGGGGGAGAT
GTTTCTGCACATGTGTGCCGCTGCCTCTGGGGTGCACATGAGTGAAAAAGCCTTT
CTGCACATGTGCAGAAAGACCTTCAGACATGTGCACCACTGCCTTTGGGGTATGC
ATGTTTGAAGGCCTTTCTGCACATGCGCAGAAAGGCCTTTCTACGCATGCGCGCT
AGAAAGGCCTTTCTACGCATGCGTGCTAGAAAGGCCTTTCCATGCATGCGCACTA
GAAAGGCCTCTCCATGCATACACAGTAGAAGACATTTCTATACATGCGCAGAAGT
AAGACCTTTCTATGCATGTGCGCTGCTTCCGGATTTCTCAAGCGGCAACTTGACA
GGTATGCTATTGTTTCTTTTATTTGAATGTGATATTCTTGTCCTAATCCAAATAATC
ACTCTGAAGTGCCATTGAAATAAATGGCCTGAACCCAAATTTCCAATGAATGGAA
TTTCACCCCTAGTGCACTCCTTGTACTGGAAAGAAAAGTACTGATTTCTGCCAATT
CCTGCTTTCCTTGGCAACCACCCCCATGTCCCACTATATTAGAAATACAGTATAGG
AGCCTCTTACACTGAGTCAATTCCTTGGTCCTTCTAGCTCACTATCATCCACATTG
AGTGGCATCAACACTCCAGAGTTTCAAAGTAAAGCAGTTCTAGACCTACGTAGAG
ATTCCAAATTTGAGGCATGTTCTACCACTGAGTTATAGATGACCTCTAGAGTCTG
AAGGACCTTTCTGTGGAAACTGAGGGTTGTAGGAGAAGGAATCAATTAGATCTAC
AACAGATTTTCTTCTGGCAGGGTCTTCTTTTGGACTTTCTCCCACAAACAGAACAG
AATCTTTCTATTTAAAGAAGGAACCCTCTGGGCTCAACCTACTCGGAGTTAAGTA
CATTATAGTACTGTATCTCACTTGGTGTTCTGCTGAAAAAGACAAAGGCCTTTCA
AGAGAGGCAGCTGTTTGCACCCTATGCCCAAACCAGTTATCATGTACTGTCTCAG
CTTGTATCATTTAAGGTAAAATGTCACAATCACGCAAGGGAACATCAAATAGCTA
TGTTGTTACCACTCAACAACAAAAGGACAAGAGAAACCAGTTGAGTAATGGAAA
AACTTACTATGACCCAGTCCCATATTTACAAATCAAAACCAAAAGTACTTGTCTTT
GTAATCACAATAAAATACAGGATAAATAACAGAGCTGTGTATTCATTTGGTTCTA
GTTTGTTACTATAAGAAGTCATATGTTAACCAAACCTATTGTTCGCTTGTCCATGG
ATTTTAAAAAAGGAACATGGAGCAAAACATTAAATAATGCAGAAGAGAACTTCA
GCTTGGTTGGGTCCTTCAGTTAGAACTGTAGAGTTACCTATTCATTTTCTTTTTCCA
GGTCTCATGAAATCTGAAGCCATTGCACATTTGAGAAGGTCCTGTTGGCTTAGAA
TAAATATGTTTGGTTTTACCAATGAAACAGTCACACAACAAGCCCTCTGAGGGTT
GTAAGATCTTCTTTATGCAAGACAGTGCAAAGTTCAACAACACAGCTACTGATTT
TATAAAGTCTTATTTACACTTAACCAAACCCCAAAGATTTTCTTGAAGCCACGTGC
CATTGTGAAACACCTCCTGCCTGTGAGGTCCATCTGTGAGTAGGAATTACAGAGA
AAGTGAAAGGAGATGGGGCTGGACCATGATCCTTCGACCACATCTCTGATATCAG
AGTGCTGAGGCTGTGACTGAGTGACTACCTTCTCTGGCTGTCTGCATGCCCTTGAA
AGCCAGTGACGCAGGGATATCACAATTGTGAGGTGAAAGTGGAGTGCCTCCAGC
ATGCTGCCAGCCATGGCTAGCTACATAACCAGAAGGAGCTGTGCTGTACGCCATG
TATGGAGACACCTGCGCTGCGGCTGGGGCAATCACTTCTGCTAGTCAGTCTTAGC
AAACAAAGATGCCAGCAGTTCCCTCTGTACTTAGACATCTGAGATCAACCATCAG
AAAGATCATTCTCCAAGCCAAGAGCATTTCTGCTAGCAGAAGATCCAGCTGCCTG
TTCCCAATCCACTACAGTTTTTGAGCGTATCAGAGAGTAAACAACAAGAACAAAC
AACAATGTTGTG

>D1_tissue_assembly_TRINITY_DN33051_c0_g1_i1_Swissprot_sp|Q2VL56|PAX9_SAGOE

GGCAGAGAACACACACTACACCTTGGGACCAGCTAAAATCCTGCAAAATGAACA
TGTTTATTGAGTTCTCCTTCACCAAACCAGAGCCCGCCTTCGGAGAGGTGAACCA
GCTGGGCGGCGTTTTTGTCAACGGGCGCCCATTGCCCAACGCTATCCGACTCCGG
ATCGTCGAACTGGCCCAGTTGGGCATCCGGCCGTGTGACATTAGCCGGCAACTGC
GCGTCTCGCACGGCTGCGTCAGCAAGATCCTGGCTCGCTACAACGAGACGGGCTC
CATCTTGCCCGGCGCCATCGGAGGCAGCAAGCCGCGGGTCACCACCCCGACGGT
GGTGAAACACATCCGGACCTACAAACAAAGGGACCCGGGCATCTTCGCGTGGGA
GATCCGGGACCGGCTTCTAGCCGACGGCGTGTGTGACAAATACAACGTGCCCTCG
GTCAGCTCCATCAGCCGCATCCTGCGCAACAAGATCGGGAATCTGGCGCAGCAA
AGTCACTACGACTCCTACAAGCAGCACCAGGGCGCCTCGCAACCCACCCTGCCTT
ACAACCACATCTACTCCTACCCGAGCCCGATCCCCACGGCCGGAGCCAAAGGGCC
TGCCCCGCCGGGCGTGCCGACCCTCCACGGCGCCGTCCATATGCCTCGGACCTGG
CCCTCCTCCCACTCCGTCACTGACATCCTAGGGATCCGCTCCATAACAGACCAAG
TAAGCGACGGCTCGCCCTACCACAGCCCCAAAATGGAAGAGTGGAGCAGCCTCA
GCAGGAGCAGCTCCGCCGCCTCGTCACCCCCGCCAGCATGAACCCCAG

>TRINITY_DN59367_c1_g2_i11_1

GAAGGAAGGAAGGAAGGAAGGAAGGAAGGAAGGAACAAACAAATGAATGGGA
AAGGAAAGGAAAGAAAGAACCTAAAAACTCTGTCTGCTCCTACTGATTGCATCTC
ATGATGACTATTCTCAGTTCCACATCTGACATGGCTAAATGATTGCAGTAGAAAA
AATGTTTTGGAAAGTTTCCCAGGAAGTATTTGTATGACATCACATATTATTTTCTT
TGCACAGCCTTAGAAATCTACCTTTGTTGGCATTGTAAATTAAAAGCATGAAATT
CTCATTAAAAAAGCAGCCTCTTATCTTGCTGTCCCAGCAGTACGGGGTAATATGG
CTAGGAAGAAACAGAGATAATTTTATTAAAGCAGTCTTTCAAAGTCTGTATTGAA
TGGTGTATTTTTACATTATTGCACTAGACTTACAAAAATACATAATATATTAATAT
CTTATAATGCTAAGAAATATGTCAATTTTCCATTTTCTTTCTTTTCATGGTTATTCT
TCAACACGGCATCTGCAAGCTTGAGAAGGCTTTCTGAAATTATATTCACAACTTT
ATTATTGTCCAAGTAGAACAGGCCATCTTGAAAAGATTATACTGTTAATGCCAAA
GGGTAAAAGAGAGCAATCAGAATCCTGGTGGCCACAAAATGATGCTTGGACAAG
AATACAAAAATGGCAGGGAAGGGGGGAGTTTAAAAAGATTTTTTTTAACATTATA
AATAATATTTGTAGCACCACCACCAATATGCCTAGGAGTGAAACATGCTTTTGTA
CAATATTGTAAACTTTACCTAAAGTTTCCTTCTTTTTTACATTCAACAAAACAGGA
ATTTTAGACATGCTTTCTTCTTTTGTTGTATGCTTGCATGGTTAAAAAAAACAATA
TAATGTGGGGTCAGGAGTCTCTGAACACAGATTAGCCAACACATTACAGTACAGT
TTTCTTTCACAGTGGTTTGGCTAATGCTGGGCATCGTTCACCTGCCTCTCTCATGC
ACAGCCACTGCAACCAGGGGTGTGAGTGTGCCCATAGGTTCTGCCATGCTTGGGC
AGAAGAACTTCCTGGTGGGATTCTGCCCTTGGCTAATTAAAAAAACAACACAAAA
TTCACATTAAACATAAGCCTCCACTTTGTTCAGATTCTTTCTATCCATTGCATAGC
TGTCAAGTATCATGAATTAGCTGGGATTTCCCATATTTTGGGGCTCCTTCCCTCTT
TTTTCTGCTGCCATCTCATATTTTGGCAGCTGGGGGGAGATGTTTCTGCACATGTG
TGCCGCTGCCTCTGGGGTGCACATGAGTGAAAAAGCCTTTCTGCACATGTGCAGA
AAGACCTTCAGACATGTGCACCACTGCCTTTGGGGTATGCATGTTTGAAGGCCTT
TCTGCACATGCGCAGAAAGGCCTTTCTAGGCATGCGCGCTAGAAAGGCCTTTCTA
CGCATGCGTGCTAGAAAGGCCTTTCCATGCATGCGCACTAGAAAGGCCTCTCCAT
GCATACACAGTAGAAGACATTTCTATACATGCGCAGGAGTAAGGCCTTTCTATGC

ATGTGCGCTGCTTCCGGATTTCTCAAGCGGCAACTTGACAGGTATGCTATTGTTTC
TTTTATTTGAATGTGATATTCTTGTCCTAATCCAAATAATCACTCTGAAGTGCCAT
TGAAATAAATGGCCTGAACCCAAATTTCCAATGAATGGAATTTCACCCCTAGTGC
ACTCCTTGTACTGGAAAGAAAAGTACTGATTTCTGCCAATTCCTGCTTTCCTTGGC
AACCACCCCCATGTCCCACTATATTAGAAATACAGTATAGGAGCCTCTTACACTG
AGTCAATTCCTTGGTCCTTCTAGCTCACTATCATCCACATTGAGTGGCATCAACAC
TCCAGAGTTTCAAAGTAAAGCAGTTCTAGACCTACGTAGAGATTCCAAATTTGAG
GCATGTTCTACCACTGAGTTATAGATGACCTCTAGAGTCTGAAGGACCTTTCTGTG
GAAACTGAGGGTTGTAGGAGAAGGAATCAATTAGATCTACAACAGATTTTCTTCT
GGCAGGGTCTTCTTTTGGACTTTCTCCCACAAACAGAACAGAATCTTTCTATTTAA
AGAAGGAACCCTCTGGGCTCAACCTACTGGGAGTTAAGTACATTATAGTACTGTA
TCTCACTTGGTGTTCTGCTGAAAAAGACAAAGGCCTTTCAAGAGAGGCAGCTGTT
TGCACCCTATGCCCAAACCAGTTATCATGTACTGTCTCAGCTTGTATCATTTAAGG
TAAAATGTCACAATCACGCAAGGGAACATCAAATAGCTATGTTGTTACCACTCAA
CAACAAAAGGACAAGAGAAACCAGTTGAGTAATGGAAAAACTTACTATGACCCA
GTCCCATATTTACAAATCAAAACCAAAAGTACTTGTCTTTGTAATCACAATAAAA
TACAGGATAAATAACAGAGCTGTGTATTCATTTGGTTCTAGTTTGTTACTATAAGA
AGTCATATGTTAACCAAACCTATTGTTCGCTTGTCCATGGATTTTAAAAAAGGAA
CATGGAGCAAAACATTAAATAATGCAGAAGAGAACTTCAGCTTGGTTGGGTCTTT
CAGTTAGAACTGTAGAGTTACCTATTCATTTTCTTTTTCCAGGTCTCATGAAATCT
GAAACCATTGCACATTTGAGAAGGTCCTGTTGGCTTAGAATAAATATGTTTGGTT
TTACCAATGAAACAGTCACACAACAAGCCCTCTGAGGGTTGTAAGATCTTCTTTA
TGCAAGACAGTGCAAAGTTCAACAACACAGCTACTGATTTTATAAAGTCTTATTT
ACACTTAACCAAACCCCAAAGATTTTCTTGAAGCCACGTGCCATTGTGAAACACC
TCCTGCCTGTGAGGTCCATCTGTGAGTAGGAATTACAGAGAAAGTGAAAGGAGA
TGGGGCTGGACCATGATCCTTCGACCACATCTCTGATATCAGAGTGCTGAGGCTG
TGACTGAGTGACTACCTTCTCTGGCTGTCTGCATGCCCTTGAAAGCCAGTGACGC
AGGGATATCACAATTGTGAGGTGAAAGTGGAGTGCCTCCAGCATGCTGCCAGCC
ATGGCTAGCTACATAACCAGAAGGAGCTGTGCTGTACGCCATGTATGGAGACACC
TGCGCTGCGGCTGGGTAAGGTGCCATCGTCGGTGCAGAGACAAAACTTCCAACTG
TTGGAAGACCGCTTGGTGCCTGGCTGTACTTGGCTTCTTGCTCCAGCGAGCCTTTG
TCCAGCCCGTTCACCGCCACGTGCTGAGCCGCCGCCGCCGCCGCCGCTGCGAAGC
TGCTCCTGCTGAGGCTGCTCCAC

HPRT

>D1_embryo_assembly_TRINITY_DN53192_c6_g1_i4_Swissprot_sp|Q9W719|HPRT_CHICK

CGCCATTTTGTGCCCCGCCGGCCTCGCTCGCTAAAGCGAAGGCGGACCACAGGCC
GCGGCAGGCCTCGCCCGGGCCTAGCGGCGGGGAGGAGGCGGCGCTGCCTGCCTG
CCTACCCGCCCGGCTACCTGGCTTTAGCCGCTCCGCCTCCCTCCTCCGAGGGGCG
GGCGTGAGGCAGGCCGGGGCCGGGCGGGCCTCCTCACAGGCAGGCAGGCGGGAG
CCGTTTTTGGCGAAGCGAAGGCGAGCAGGCGAAGGGAAGGTATCGCGGCGGCGC
CCATGGCCGGCCCGAAGACCAACGCCGAAGGCAGCTCCAGCCGCGGCTCCTGCA
TCGTGATTGAGGATGATGAACAGGGTTATGACTTAGACTTGTTCTGCATACCAAA
ACATTATGCGGAAGATTTGGAAAAGGTTTACATTCCCCACGGACTTATTATGGAC
AGGTTGGTTGGACAAGGCAGGAACATTAAAAACCAGTTGTTTCCATGCAGGACA

GAAAGATTGGCACGGGAGATAATGAAAAGCATGGGAGGACATCACATTGTCGCT
CTCTGTGTACTTAAGGGGGGCTATAAATTCTTTGCTGACTTGCTGGATTATATCAA
AGCCCTGAACCGAAACAGTGACAAGTCTATTCCTATGACTGTTGACTTCATCCGA
CTCAAGAGCTACTGTAATGACCAGTCAACGGGTGAAATAAAAGTAATTGGCGGT
GACGATCTCTCAACTTTAACTGGGAAGAACGTCTTAATTGTAGAAGATATAATTG
ACACTGGCAAGACAATGAAGACCCTGCTCTCTCTCCTCAAACAGTACAATCCAAA
GATGGTAAAGGTAGCCAGCCTGCTGGTCAAAAGGACACCGCGAAGTGTAGGATA
CCGGCCCGACTTTGTCGGATTTGAAGTGCCGGACAAGTTTGTGGTGGGATATGCT
TTAGATTATAACGAATACTTCCGGGATTTGAATCACATTTGTGTGATAAGCGAGA
CGGGAAAGCAGAAGTACAAAGCGTGACATTGATGCTTTCAGTGCGTTTTGGAGCT
CTCGTTTTCCCATTGACTTCTTCGGCTTTAGTCTCCAGCTTTGAATCATGGGATGTT
CCAGCCGTGTCGTCATTTGCTTGCTATAATTTATTGCATGTACAGAATAGAAATCT
TGTCTTGTTCATTTATGTCGGGGAATTTTTTTTGCAGGGAGTGCAATTATTCACCT
CCTCCCTCCCCATTCTTTTTAAATTTGCACTATGAGTCTACTAATTCTTGCGCCCCC
CCCCCATCTCCCTCCCAGTAAGGGATTTTGCTGTGTGTTGGTCCCAAGCTTAGCTC
TTCCCCAAAGTCATTACATTTCTTCCCCTTCCTTTTAAACCTCATCTACTCAGAAC
AGAAATAGTTATAAGTACTGTATATGTATAAGACATTTTAAGAGGGAAAATTATA
TTAGTAATATTTTTTTAAAGAAAACAAGTAATTTAAGTTTTATATTGTAATTAATT
CAAAAATTGGGATGGTTGTTGGATAGCTGCTGCGGGGGGCTGGTGTACAAAAAC
AACGTGAAGGGGCAGCTGTCCAGTAAATGCTGCGGTCTTGAGTATGAACACTGTT
CTGCTAAACTGTTTCCTCTGGAGTTGCCTTACTTGCCTCTTAGAGGGTTTTTTAAA
AAGGTTATTTTGTCCTGAAAAGAGAGAGCAAGTGACCGAATCGAATGTCGAAGC
AGCTTCCGAGCTTTCCGACTCCTGCAGGCATGGTGATGTCACAGTCTACACAGGG
AAAGAGTCTTCTCAAAGCAGAGGAGAGCAGGAGTAGAGTTCCATCAGGCATGCT
CAGTAGCAGTTGCTGGTCGGCCGGGGTGTCCAAACCTTTCGGGCCGTGGGCCGAT
CATGCGATTCAGTGGGCCGGTCACATGGTTCCTCCCACCGGAAGCGGGAGTAAAA
CACACCTTGCAATCGGTTTTGCAACAGGATGAGATAGATTTTAAGCTGCCATTTCT
CTGCTTAAACTGGGAGCACCCAAACTACAGGCCATGGGCCAGATCCAGCCCTTGA
CACCAATTCATCTGGCCCACACTACCCTCATCAATCCTGGAAG

>D1_embryo_assembly_TRINITY_DN53192_c6_g1_i8_Swissprot_sp|Q9W719|HPRT_CHICK

GCAAAATCCTCATTATTAGACACCAGAATGCACAGAACATTGTTTATATTCCCAC
TTTTTGTCCCTGCTTCTCTATGGCAGCAGATCACAGAAACAAAGTACCCTAGGCC
GTGATGGTGAACCTTGGCACTCCAGATGTTCTTGGACTTCAATTCCCAGAAGCCT
AAGTCAGTTCCTCACATTGGGAGCCACTGGATGGGGCGGGAGGGCTTTCTTGCAT
ATTCTGTCCACAAGGCACAGTCCCGTCTTCATATTTAATGTAGGGAACCTTAAGT
GTTGCTATCCTTTTCAACGCAACCTTCCCCCCTATGTTTGTGACTGTCTCAGAAAT
AGTCCATTCTTTGGTTTGTTTGCTTGACTTTCTGTACATTTTTTTCTAATTAACATT
TCATCTTATTTCCTCTCTCAAACTAGAATGACCAGTCAACGGGTGAAATAAAAGT
AATTGGCGGTGACGATCTCTCAACTTTAACTGGGAAGAACGTCTTAATTGTAGAA
GATATAATTGACACTGGCAAGACAATGAAGACCCTGCTCTCTCTCCTCAAACAGT
ACAATCCAAAGATGGTAAAGGTAGCCAGCCTGCTGGTCAAAAGGACACCGCGAA
GTGTAGGATACCGGCCCGACTTTGTCGGATTTGAAGTGCCGGACAAGTTTGTGGT
GGGATATGCTTTAGATTATAACGAATACTTCCGGGATTTGAATCACATTTGTGTG
ATAAGCGAGACGGGAAAGCAGAAGTACAAAGCGTGACATTGATGCTTTCAGTGC
GTTTTGGAGCTCTCGTTTTCCCATTGACTTCTTCGGCTTTAGTCTCCAGCTTTGAAT

CATGGGATGTTCCAGCCGTGTCGTCATTTGCTTGCTATAATTTATTGCATGTACAG
AATAGAAATCTTGTCTTGTTCATTTATGTCGGGGAATTTTTTTTGCAGGGAGTGCA
ATTATTCACCTCCTCCCTCCCCATTCTTTTTAAATTTGCACTATGAGTCTACTAATT
CTTGCGCCCCCCCCCCATCTCCCTCCCAGTAAGGGATTTTGCTGTGTGTTGGTCCC
AAGCTTAGCTCTTCCCCAAAGTCATTACATTTCTTCCCCTTCCTTTTAAACCTCATC
TACTCAGAACAGAAATAGTTATAAGTACTGTATATGTATAAGACATTTTAAGAGG
GAAAATTATATTAGTAATATTTTTTTAAAGAAAACAAGTAATTTAAGTTTTATATT
GTAATTAATTCAAAAATTGGGATGGTTGTTGGATAGCTGCTGCGGGGGGCTGGTG
TACAAAAACAACGTGAAGGGGCAGCTGTCCAGTAAATGCTGCGGTCTTGAGTAT
GAACACTGTTCTGCTAAACTGTTTCCTCTGGAGTTGCCTTACTTGCCTCTTAGAGG
GTTTTTTAAAAAGGTTATTTTGTCCTGAAAAGAGAGAGCAAGTGACCGAATCGAA
TGTCGAAGCAGCTTCCGAGCTTTCCGACTCCTGCAGGCATGGTGATGTCACAGTC
TACACAGGGAAAGAGTCTTCTCAAAGCAGAGGAGAGCAGGAGTAGAGTTCCATC
AGGCATGCTCAGTAGCAGTTGCTGGTCGGCCGGGGTGTCCAAACCTTTCGGGCCG
TGGGCCGATCATGCGATTCAGTGGGCCGGTCACATGGTTCCTCCCACCGGAAGCG
GGAGTAAAACACACCTTGCAATCGGTTTTGCAACAGGATGAGATAGATTTTAAGC
TGCCATTTCTC

>D1_tissue_assembly_TRINITY_DN45027_c0_g1_i2_Swissprot_sp|Q9W719|HPRT_CHICK

CACAACGTTTTTGTTTAAAACTTTCTTACGTCTTTCAAGTGCCACAAACGTTTTTG
TTTAAACTTTATTATCCTCGTGTGAGGAAGGATGGCGACCCCCACCCACTTTCTTC
GGGGTTGGGGATTTGAGTTTTATACATTTGGGCCTGAAGAAGTGACGGTTGTTGG
CGCCCTTCTCCTCCCCTCACAGGGGCTCCCAGGGCCGCCATTTTGTGCCCCGCCGG
CCTCGCTCGCTAAAGCGAAGGCGGACCACAGGCCGCGGCAGGCCTCGCCCGGGC
CTAGCGGCGGGGAGGAGGCGGCGCTGCCTGCCTGCCTACCCGCCCGGCTACCTGG
CTTTAGCCGCTCCGCCTCCCTCCTCCGAGGGGCGGGCGTGAGGCAGGCCGGGGCC
GGGCGGGCCTCCTCACAGGCAGGCAGGCGGGAGCCGTTTTTGGCGAAGCGAAGG
CGAGCAGGCGAAGGGAAGGTATCGCGGCGGCGCCCATGGCCGGCCCGAAGACCA
ACGCCGAAGGCAGCTCCAGCCGCGGCTCCTGCATCGTGATTGAGGATGATGAAC
AGGGTTATGACTTAGACTTGTTCTGCATACCAAAACATTATGCGGAAGATTTGGA
AAAGGTTTACATTCCCCACGGACTTATTATGGACAGGACAGAAAGATTGGCACGG
GAGATAATGAAAAGCATGGGAGGACATCACATTGTCGCTCTCTGTGTACTTAAGG
GGGGCTATAAATTCTTTGCTGACTTGCTGGATTATATCAAAGCCCTGAACCGAAA
CAGTGACAAGTCTATTCCTATGACTGTTGACTTCATCCGACTCAAGAGCTACTGTG
TTCCCTGGGAGGAGTTTGGAATAGAGATGGGGGCTTTCGCTACACCAAGCTTTGG
GAAAACTTCTTCCCAGATAGCCAGGAGGGAAGGAATCGCCACTCCAGATTGCCTG
CTCCAGAGTAAATAAAGCTGGCCTGCATCAGACGGAGAGAATGACCAGTCAACG
G

MSX1

>TRINITY_DN55566_c3_g1_i5_1

AGACATAAGAGAAAACAAAACAAAAATAGATTTTACAAATACAATCTACAAGAT
GCAAACACAAACTAATTATGCAATCGCACTACAAGCATAATGACATAACATCAA
CAGTCACTTCAAATATACAGTACCTCTCACTTTTCAACTTAACTTCACAACCTTTC
TTCTATCTTACCACTTTATATTTTTAACACACTGTCTTATTGATATTATATCCATTT

ATCAAAAATAATAATACAAAGAGAAAAAAAGTCATACAAAAAAAATAAAAATAT
GGAAAAATTCACACACCCTTTGACCCTTTACCAGAAAAAGACAGCCTGAAAACAT
TTTTTTTTCATAAATAAGAACCGGGGGTGGGGGAGTGGTTCAGATAACTTAGTTT
TATATTATTAAAGTAATTCCCAACTGCCACTTTCAAGCTCTTACTATTACTTAATC
TCCTTAAATCCTCCCTCAGGAGCAACCAAGGATCAATTTGGCTGATAAATTACAT
GTAATGGCTCTCATTAAAAAGGTGGGATCAGGTGTGCGAAAAATGGAGGAGACA
AATATTGAAATACATTATGATATAGCACTGGATGAAAGGATTTGGCGGCAAACA
GTGAGGACAATTACAAAAAGATTGATAGAAAAAGGGTTAAATTATAATTGGAAA
TTCCCAAAGCAACTAAGTTTCTTTTATTCGGGCAATCGCTTTTTCATCAACCAGAG
TTCTTCTGCACTGGACATCATGGTAAAGGCAAAATTGTTGGATAGCTGAACTAAA
GAATGGGAGATTCTGAAACAGGAATTGATAATGTCTGTAAATTTGTACAGTCCTA
ACTCAACAAAAAAATATTAACAGAGCAATTTAGAAAGGGGCAATTATATTGTTTT
GTTATTAAAAGACCTTATAAATACATTTCTTATTTTCCTGTTTGCCTGATTTCCAA
AGAAATATGAATACCAAAGGTATTCATTGCAAATTGATTAAAGAATTTCTTAGGA
AAAACTGGCAGATTTTATGTTGGATGTGATGTTTCGCATTCAGTCTGAAGACAAA
AGTGGATGATACCTTTAAGAGGCCACTAAGATCTCATATAGGGAGTCTTGTTTTC
AACTCTACATGAGTCTTTGTCAGACTGGGTATTAAAGAGTGGGAGGGGGCAGGA
AAAAAATACAAACATTTCCAAATCCATGGCCAGACATGCAAGGCAGCTTCCTCAT
TTAAAGCAAATTCCAAAATGAAAGCTGCAGAATCCTCTCAGACGTGGTAGGTCGT
CTCAGACTTCACCTCTGGCGATGTGGATTAGCCTTGCATCCCTTGTATCGGAACAC
ATGGATCCTTTTGTGGCTTCTGGGAGTCTTTTCTTCATATCGAATGATTTAGGATG
TCAGTGGAAGCTCCAAGTGCCATTCTTTAGATTTGTAACATAGCCATTATTTGCTG
CTGCCCCGTGGTGATGGTGGTGGTGGGGCAGCTGCTAAGTGAGGTGGTACATGCT
GTAGCCCACGTGTGCTGCGTAGAGTCCCACAGGGGTGACAGGCAGGCCGGTGGT
GGCACGCTGGAAAGGGCCAGACGAGGCGTACAAGGAGGCGCCAGCCATGGCCG
GTCCACCCAGCGGGAAGGAGAGGCCAAAGGCGGCGGGTGGCAGCAGGGGCTTG
GCTGCCATCTTGAGCTTCTCCAGCTCGGCCTCCTGCAGGCGCTTGGCCTTGGCACG
CCGGTTCTGGAACCAGATCTTGACCTGCGTCTCCGTCAGGCTAAGCGAGCTGGAG
AACTCGGCCCGCTCGGCGATGGACAGGTACTGCTTCTGCCGGAACTTCCTCTCCA
AGGCCAGCAGCTGTGCCGTCGTGAAGGGCGTCCGGGGCTTGCGGTTGGTCTTGTG
CTTCCGGAGGGTGCAGGCCGGGGGGCTGGCGTGCCTTGGAGGCGGCGGCGGAGG
GGAGAAGCGGGGGCTTTGCATCCAGGGCGTCCGCTCCGTTTTCTCGGGGCTTTCC
GCTTTGAGCCGCGCCTCGTCGGGCAGCTTCGCTAATCCCCCGACGGCAAAATGGC
TGGCCACGGCGAGGGGGGAGGCCGGCCCTTCCGGCACCGAGGCGGAAGACCCCC
CCATCCTGGGGCTCAGGGGGGTGCTCCGATGGGCCAAGGGCCCGCCCAGATCTG
GCCCGCTGGCCGAGGGGTCTCGGGCGCCGCCGCCGCCGGGCTTCTTGTGATCGGC
CATGAGCGCCTCGACACTGAAGGGCAGCAACGCCGCCGGGGCTGCCGCCTTGGG
CTTGCCGCCTTCCTCCTCCTCCTCTTCCTCCTCCTCCTCTCCCCTCATGGCGCC
CTCCTGCCTTGG

>CL19192Contig1_1
CAAATTAAATTGAATTTACTCTCAAGTAGCTATGGAGGGAAACCTTTTATCAAAT
ATTTGTAAGCCAGTTTTATTTCCTATAATACTAACAGTACAATCCTAGACATGCTT
TGTCAGGCAAATTGTTCAGCCTAGTAGGGCACTTTCCAAGACATAGGGAATTATA
GTAGAATTTGAATTTTGGTTTAGCCAAATGAAAATTCACTTAAAGGCAGACTTGC
AGAATCTATTATTCCAGCACAATGGCTCCTGGTCACTTCAACCAAGTGTCTCTAA
AACTGAGATTGAAGTGGGAATGCTTCCATGTAAAGGCTATTGTATAATATCCATT

ATGCTGATGGATGTGTCTGTATATTCTCATAAGAATAAGAAAATGTAATGGCATT
CCAGGACAAAGCAAGAAACGCATTTATGTCAACTGGACTTATCTTCCAAATGGAT
AGTCTTGATATGGGTGGCTCACGGATACAGTGTATCTCACTCCAAAATTGTTGTC
ATAAGTGTACCTCATTTCTTTGGGATGTCCTACTTTGTAGAAAAAATGTAGATATT
TGAAGGGCAAGGTTAATTATATGCTAGGAATTAGGTTCCTTGCAATCAATGGAAT
TTTCTTCCTTCAGAGGAACTAGAATCCTTTTCAGGTTGACCCATACAAACTGCAGT
GTGTCAATTCTTTGCTGGTGGATTTAATTTAAGAGCCAGATAATAAACAGTGTCG
ATCTGAGACCAAGCAAATACATTCACTTCCCTAACAGAGAGATATCTGGGACTAA
AGTGAAACCCTTTAGATCTTCGATTTGGGACCAAGCAAATACATTTACTTCCCTA
ACAGAGAGATCCTGGGACTAAAGTGAAACCTTTAGATTTTCGTACTTGGAAGGCA
ACGTCTCGCCCCGTCAAGGTCGTTTAAAATTAATTTATTTATCAAATATAGCTATT
AATATAAATGATAATAAATTTTCAACTGTACAGTATATTACACAAGAACCCTTTC
TGAAGGCATGGGTAGGAGAACAGGAGGCAAAGAGCTGGCAGGGGGGGCGTGTTT
GGGGGGTTTGAGATGGTGGCATCCATACAGGCCTGACCCAGAAAGGATGTCCAG
GTCCCTTGAGAGCGACCCAAGGGCCATGTGGGGCTCGCTCTCTGCCACTTCTTCC
CCAGCTGTCTGGTGACCCCCTACTCCAAGAAGGGCCGGGATGGTATGAGCTTTTC
CGTTTGTGCCTGACCTGCTGCCCCGTGGTGATGGTGGTGGTGGGGCAGCTGCTAA
GTGAGGTGGTACATGCTGTAGCCCACGTGTGCTGCGTAGAGTCCCACAGGGGTGA
CAGGCAGGCCGGTGGTGGCACGCTGGAAAGGGCCAGACGAGGCGTACAAGGAG
GCGCCAGCCATGGCCGGTCCACCCAGTGGGAAGGAGAGGCCAAAGGCGGCGGGT
GGCAGAAGGGGCTTGGCTGCCATCTTGAGCTTCTCCAGCTCGGCCTCCTGCAGGC
GCTTGGCCTTGGCACGCCGGTTCTGGAACCAGATCTTGACCTGCGTCTCCGTCAG
GCTAAGCGAGCTGGAGAACTCGGCCCGCTCGGCGATGGACAGGTACTGCTTCTGC
CGGAACTTCCTCTCCAAGGCCAGCAGCTGTGCTGTCGTGAAGGGCGTCCGGGGCT
TGCGGTTGGTCTTGTGCTTCCGGAGGGTGCAGGCCGGGGGGCTGGCGTGCCTTGG
AGGCGGCGGCGGAGGGGAGAAGCGGGGGCTTTGCATCCAGGGCGTCCGCTCCGT
TTTCTCGGGGCTTTCCGCTTTGAGCCGCGCCTCGTCGGGCAGCTTCGCTAATCCCC
CGACGGCAAAATGGCTGGCCACGGCGAGGGGGGAGGCCGGCCCTTCCGGCACCG
AGGCGGAAGACCCCCCCATCCTGGGGCTCAGGGGGGTGCTCCGATGGGCCAAGG
GCCCGCCCAGATCTGGCCCGCTGGCCGAGGGGTCTCGGGCGCCGCCGCCGCCGCC
TCCGGGCTTCTTGTGATCGGCCATGAGCGCCTCGACACTGAAGGGCAGCAACGCC
GCCGGGGCTGCCGCCTTGGGCTTGCCGCCTTCCTCCTCCTCCTCCTCTTCCTCCTCC
TCCTCCTCCTCTCCCCTCATGGCGCCCTCCTGCCTTGGCTTGGCAAGCGGCGCCTC
TTCTGCTCCTCTCGCCCCGCTCGGGACCGAAGAAGTCATGGCCAGCCCTTCTGGG
GCCACCCGCCGCCGCCGCCGCCGCTCATGGAGGTGGGGGCGCAGCGCAGCCGTG
ACCGTCGGTCGGTCGGAGCAAGTCCTCCGCGCTCCTCCACGCCGCCCGCGCCTGC
GGGACACACTTT

>CL3504Contig1_1
GGCATGTGCAGCAGGGAAGGCCTACCTGCCCTGCGCATGCCCAAAAGGCCTTCAC
CTCCAGGTTCGCTTTCCTGACGGGCCTGGGCCGGGTGGAGGGCTCTAATAGGCTC
GTGCCTGCCAGGGAAGGTGAGCTGGGTGCCCCCTTCCCTGGCGGGCCTGGGCTGG
TCGGAGGACTCCGATTGGCCCAGGGAAGGCGAGCCCGGCACCCCCCTCCTTGGCG
TTCCTGGGCTGGGTGGAGTGCTCTGTTTCGCCTGCTGGGGAAGTCAAGGTGCCCT
CTTCCCTGGTGAGCCTGGGCCTGTGTGTGTGAGAGAGAGAGAGGAAGGGGGGAG
TGGGCCAGACAGGGTCCGGGCAACACCAGGTAGCCCTGCTAGTCATGCCTATAAT
TTACAATCTGTACGTGAAAGACAAATTCTGAGTTCAGGTTTGGAATCAGCACACT

CGATATTATATAAATCACATGTATTATTCCCAGTAGCAAAATTGTTGTTGTGCAGT
GAAATCAATGCTCCCAATGGCACTCGGTGTGTGTGTTTGTGGCTATACTGCTGGA
TAGATTTGTCCTACAGAAGGAGAGCTGTGGGTTGGAGGGGGAGACCCCACATCT
GGTACAGCCTGGAGCGATTCCAGCCACATAATTTCCCAGGTTGTTCTTTTAAAGC
AGCTATCCACAGCCAGAGCAGGGAAGGGGCTCCATTGAACCCAGATGTGGGGAA
AACCCCTCTGTGCCCTTCAAAGGTTGGACTGGAACGCCCCTCTCCATCTCTGGGTG
GGTGGGGGGCTTTTCCAGTATAATAGTGTATCAGCTCCTGGGGCTGCTGTGGCTC
AGTGTTTATGAAACTCACCTTGTTAGCTGGAAAGCCAGCAAGTTGGCAGTTCAAG
ACCTGGGTACTGCATGACGGATTGAGTGCCCATCTCTGTCCCAGCTCCTGCTAAC
CTAGCAGAGCAGAAGCAAGAAAATGCACATAGATAAATAAGGACCACTTTAGTG
GGGAAAAGATCTCGGGGACATGAAAGGAGCCATATTGGGTATCCCCTTAGTGGA
GTTGCTCCAGCAAAAAATCCTTTCAACCCAGAATAATTTCTGACACGAAAATTAT
TACTGTATTATTATTATTATTATTATAGCTCTTGGTAGCTCCATGTAATTCCATTGT
TCTTTGAGCCCTTCACTCACCCACGTAGTTCAATTCAACCATGTACCACCTCCCGT
CCTTTTTTTTCAATCACAGCATGGACTCTGTTTCTAGTTTCACATTTGTGTAGGAGT
TTTGTAAGGGTGGGGTGGGGAGTCATTTCGGG
