# Supplementary figures and images for: Polarized Sonic Hedgehog Protein Localization and a Shift in the Expression of Region-Specific Molecules Is Associated With the Secondary Palate Development in the Veiled Chameleon
Source: Front Cell Dev Biol. 2020 Jul 28;8:572. doi: 10.3389/fcell.2020.00572 (PMC7399257; doi:10.3389/fcell.2020.00572)

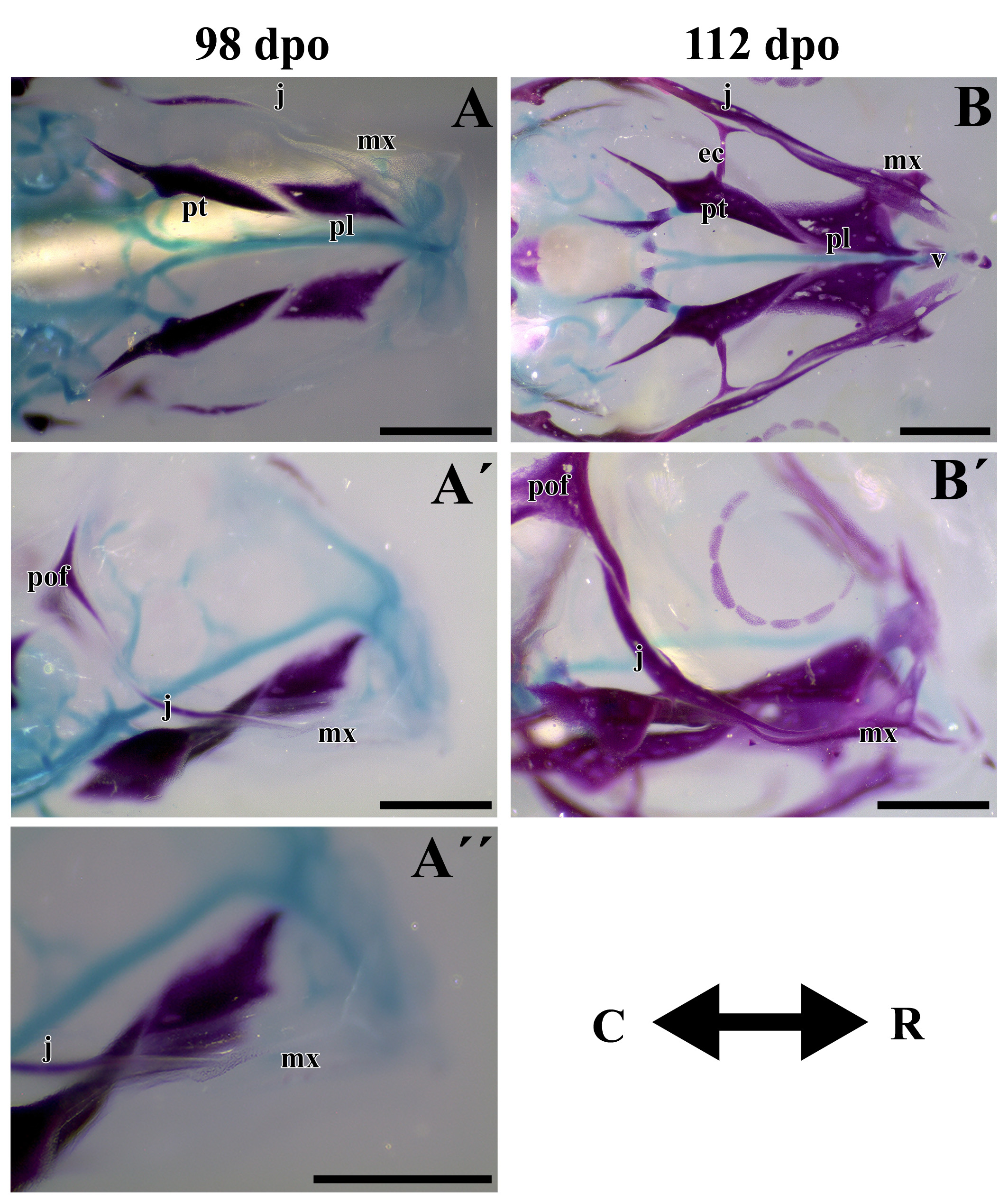

Supplement: FIGURE S1 — Detailed palatal and lateral view on chameleon skull at pre-hatching stages. Palatal view on the chameleon head at earlier stage (98 dpo) and at later stage (112 dpo) (A,B). Lateral view on the embryo at 98 dpo stage demonstrate ossification centers of maxillary bone (mx), jugal bone (j), and post-orbitofrontal (pfo) bone (A′,A″). Lateral view on the embryo of the 112 dpo old with focus ossification centers of maxillary bone (mx), jugal bone (j), and post-orbitofrontal (pfo) bone (B′). Pictures orientation: left (caudal, C), right (rostral, R). Scale bars: 1 mm. [file Image_1.JPEG]

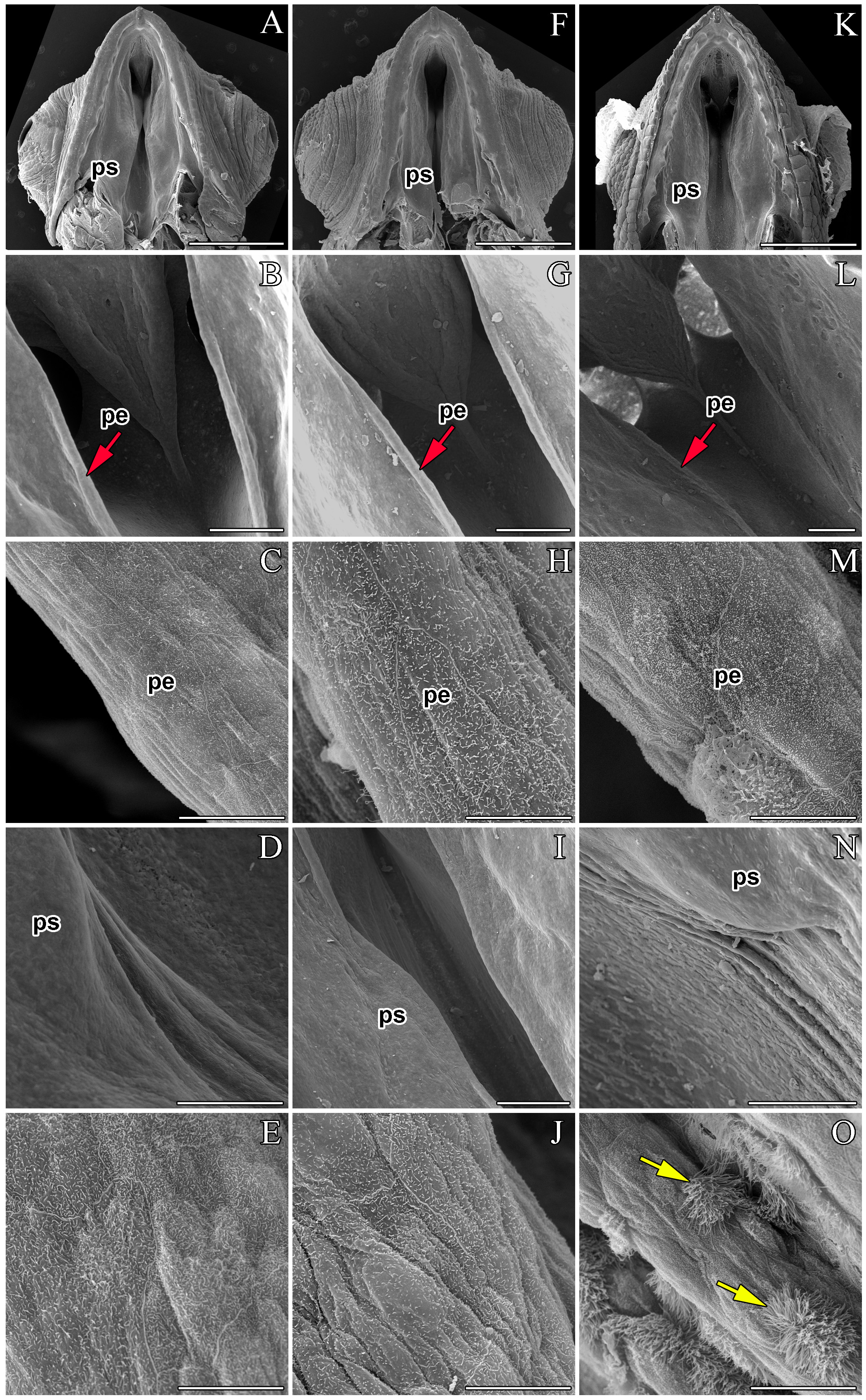

Supplement: FIGURE S2 — Palatal shelves morphology and surface structures arrangement of chameleon embryos in scanning electron microscope. Palatal view on prehatching stages of chameleon at age 17 weeks (weight of embryo 0.36 g), (A–E), at age 18 weeks (weight of embryo 0.42 g), (F–J) and at age 20 weeks (weight of embryo 0.53 g) (K–O). Low power view on the palatal shelves (ps) (A,F,K). Higher magnification on the rostral areas of the palatal shelves with magnification on the palatal edge (pe), (B,C,G,H,L,M). Higher magnification on the caudal areas of the palatal shelves with magnification on surface structures highlighting motile cilia in the oldest embryo (yellow arrow), (D,E,I,J,N,O). Scale bars: (A,F,K) – 2 mm, (B,G,L) – 200 μm, (C,H,M) – 10 μm, (D,I,N) – 200 μm, (E,J,O) – 10 μm. [file Image_2.JPEG]

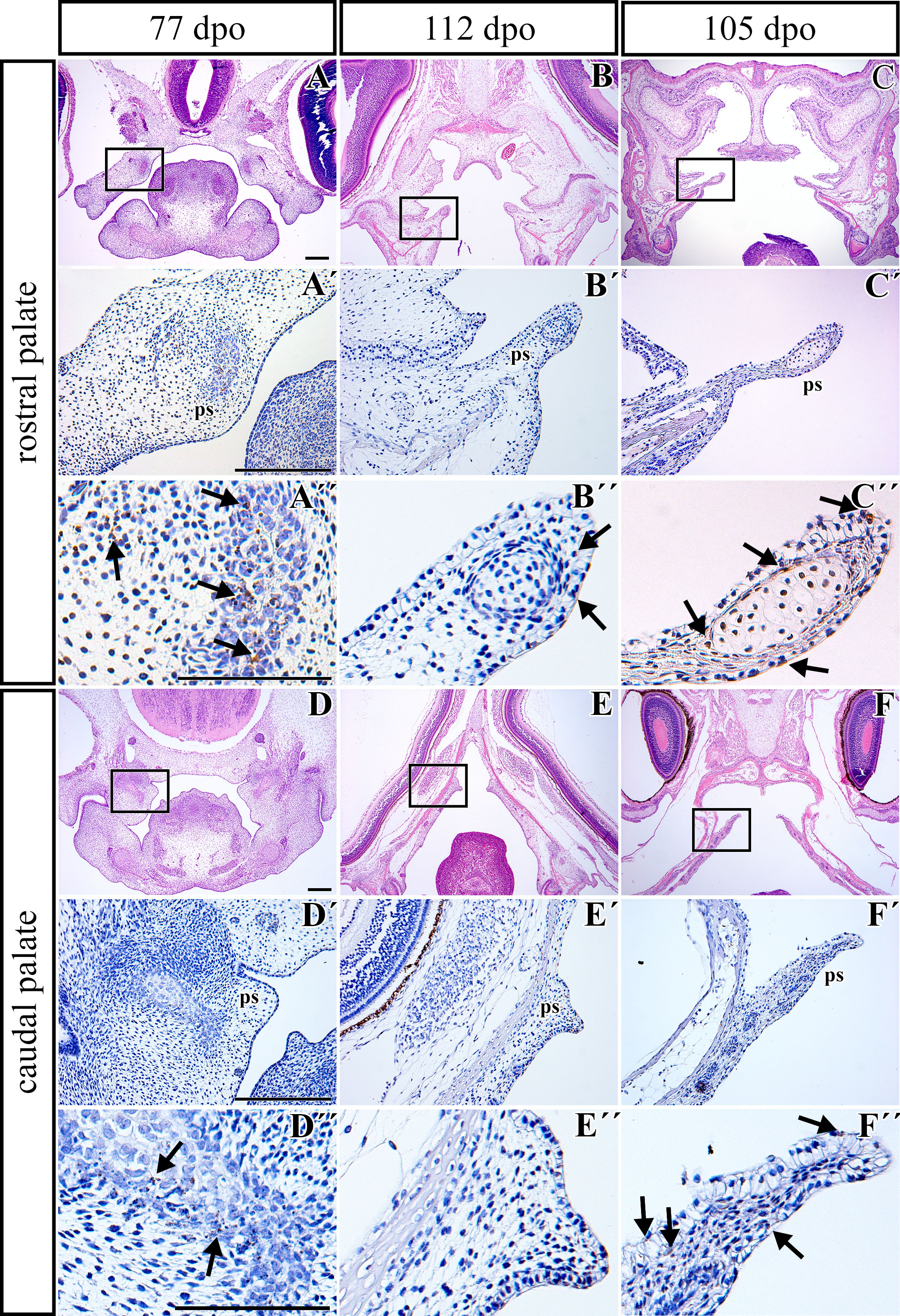

Supplement: FIGURE S3 — Distribution of apoptotic cells in chameleon embryos. Cell death in the palatal shelves of three different pre-hatching stages of the veiled chameleon. HE stained frontal head sections in the lower power from rostral (A–C) and caudal (D–F) areas of the palatal shelves. Higher power view (details from black rectangles) of TUNEL-positive cells on transversal sections through head in rostral (A′–C′) and caudal (D′–F′) areas of the palatal shelves. Details of the palatal shelves with black arrows pointing on TUNEL-positive cells (brown) in either rostral (A″–C″) or caudal (D″–F″) regions of the palatal shelves. Nuclei (blue) are counterstained with Hematoxylin. Ps, palatal shelf. Scale bars: 200 μm, details: 100 μm. [file Image_3.JPEG]

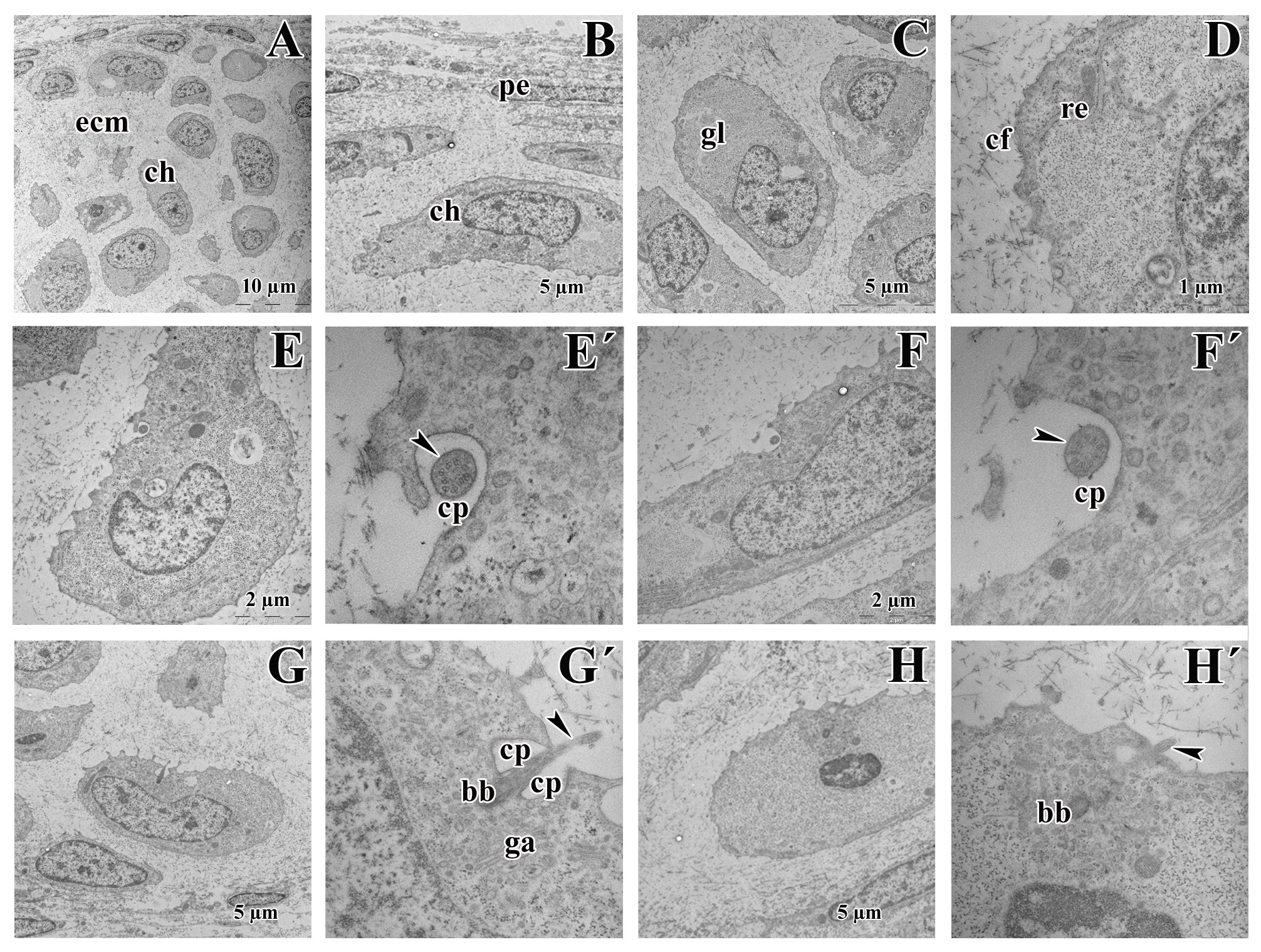

Supplement: FIGURE S4 — Ultrastructure of the palatal cartilage and primary cilia morphology in chameleon embryos. The rudiment of the palatal cartilage was present closely adjacent to nasal part of the palatal shelf and chondroblasts expanded into tip of the palatal shelf. Chondroblasts located on the cartilaginous periphery were flattened, centrally situated cells were oval or round-shaped. Primary cilia were frequently observed in the epithelium, mesenchyme or in the palatal cartilage (arrowheads). In chondrocytes, they were embosomed by membranous structuresof Golgi apparatuses. Bb, basal body; cf, collagen fibrils type II; cp, ciliary pocket; ch, chondroblast; ecm, extracellular matrix; ga, Golgi apparatus; gl, glycogen; pe, perichondrium; re, rough endoplasmic reticulum. [file Image_4.JPEG]

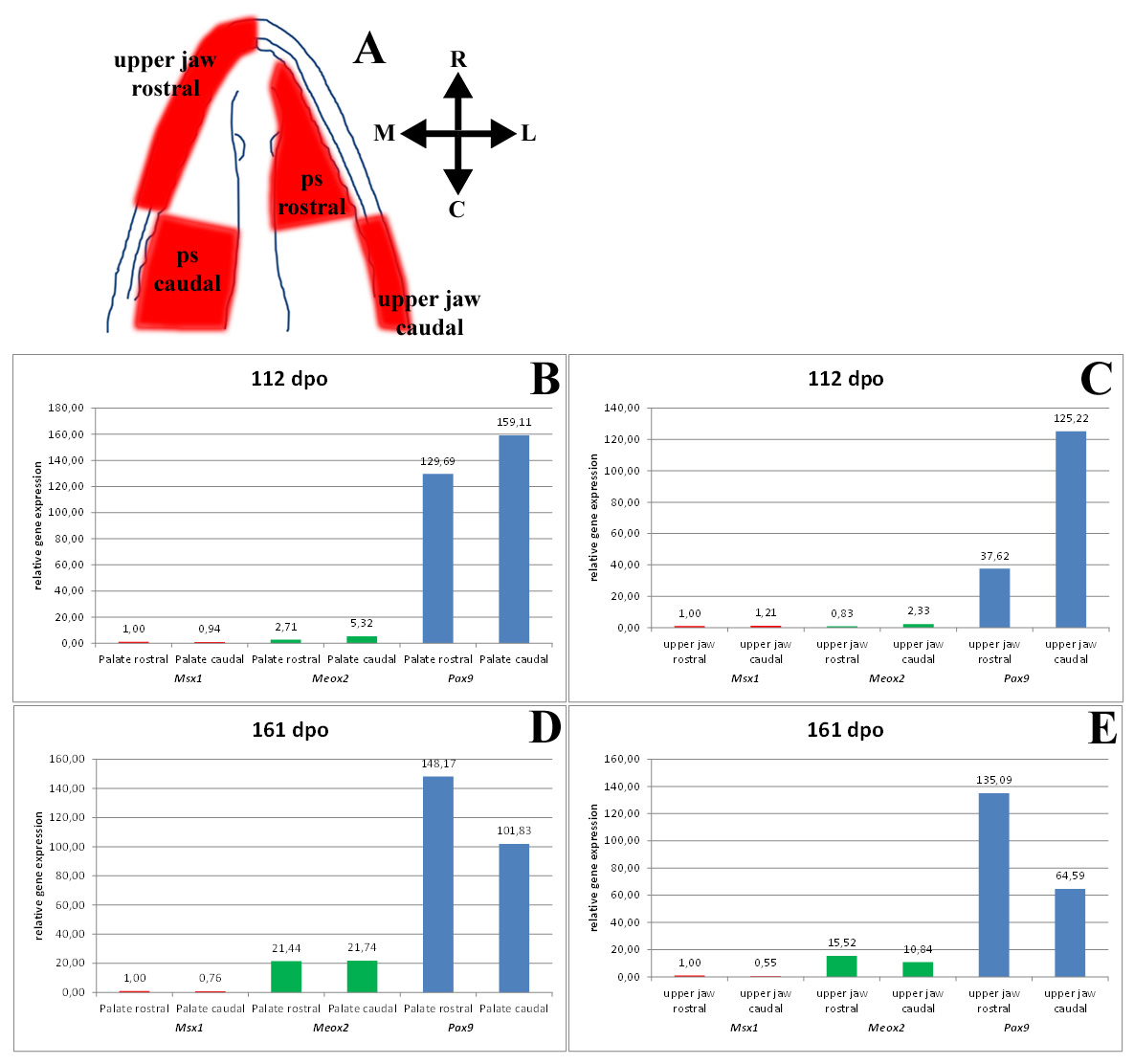

Supplement: FIGURE S5 — Gene expression analyses. Labeled areas from which tissues were collected for QPCR analyses (A). Comparison of gene expression of Msx1, Meox2, and Pax9 in the palatal shelves and in the upper jaw during pre-hatching development of the veiled chameleon. Gene expression comparison between individual genes in the palatal shelves of the earlier stage (B) and later stage (C), and in the upper jaw of the earlier stage (D) and later stage (E). Gene expression is shown as relative gene expression. In each group, gene expression of Msx1 in the rostral tissue was used as a control for comparison. [file Image_5.JPEG]
