# Supplementary material for: Polarized Sonic Hedgehog Protein Localization and a Shift in the Expression of Region-Specific Molecules Is Associated With the Secondary Palate Development in the Veiled Chameleon
Source: Front Cell Dev Biol. 2020 Jul 28;8:572. doi: 10.3389/fcell.2020.00572 (PMC7399257; doi:10.3389/fcell.2020.00572)
Supplement: TABLE S1 — List of contigs used for the primer and probe synthesis. [file Table_1.pdf]

Table S1: List of contigs used for primer and probe synthesis

gene	obtained from	name of contig	best percent identity	best percent identity to
MEOX2	C.calyptratus_annotated_meta_transcriptome	D1_embryo_assembly_TRINITY_DN59016_c3_g5_i2_Swissprot_sp|P50222|MEOX2_HUMAN	87.38%	XM_020782199.1
PAX9	C.calyptratus_annotated_meta_transcriptome	D1_tissue_assembly_TRINITY_DN47668_c3_g2_i9_Swissprot_sp|Q2VL51|PAX9_LEPED	79.07%	XM_028720443.1
PAX9	C.calyptratus_annotated_meta_transcriptome	D1_tissue_assembly_TRINITY_DN33051_c0_g1_i1_Swissprot_sp|Q2VL56|PAX9_SAGOE	88.51%	XM_028720443.1
PAX9	C.calyptratus_embryo_transcriptome_fpkm_1	TRINITY_DN59367_c1_g2_i11_1	79.07%	XM_028720443.1
MSX1	C.calyptratus_tissue_transcriptome_fpkm_1	CL3504Contig1_1	86.46%	XM_020805799.1
MSX1	C.calyptratus_embryo_transcriptome_fpkm_1	TRINITY_DN55566_c3_g1_i5_1	86.22%	XM_020805799.1
MSX1	C.calyptratus_tissue_transcriptome_fpkm_1	CL19192Contig1_1	86.13%	XM_020805799.1
HPRT	C.calyptratus_annotated_meta_transcriptome	D1_embryo_assembly_TRINITY_DN53192_c6_g1_i4_Swissprot_sp|Q9W719|HPRT_CHICK	82.34%	XM_020810323.1
HPRT	C.calyptratus_annotated_meta_transcriptome	D1_embryo_assembly_TRINITY_DN53192_c6_g1_i8_Swissprot_sp|Q9W719|HPRT_CHICK	81.18%	XM_020810323.1
HPRT	C.calyptratus_annotated_meta_transcriptome	D1_tissue_assembly_TRINITY_DN45027_c0_g1_i2_Swissprot_sp|Q9W719|HPRT_CHICK	91.07%	XM_020810323.1
