# Supplementary material for: Polarized Sonic Hedgehog Protein Localization and a Shift in the Expression of Region-Specific Molecules Is Associated With the Secondary Palate Development in the Veiled Chameleon
Source: Front Cell Dev Biol. 2020 Jul 28;8:572. doi: 10.3389/fcell.2020.00572 (PMC7399257; doi:10.3389/fcell.2020.00572)
Supplement: TABLE S2 — List of the primers for PCR and QPCR. [file Table_2.pdf]

Table S2: List of primers for PCR and QPCR

gene	forward	reverse
Msx1	5`-ACGTGTGCCGCATAGAGTC-3`	5`-ATTTTGCCGTCGGGGGATTA-3`
Meox2	5`-ACGGCTTTCACCAAAGAGCA-3`	5`-CAAGGCACGTTTAAGGTGTGG-3`
Pax9	5`-AGCACCACCACCAATATGCC-3`	5`-AGAGACTCCTGACCCCACAT-3`
Hprt1	5`-ACAGAAAGATTGGCACGGGA-3`	5`-AGTTGAGAGATCGTCACCGC-3`
